# Discrete Ti−O−Ti Complexes: Visible‐Light‐Activated, Homogeneous Alternative to TiO_2_ Photosensitisers

**DOI:** 10.1002/chem.202001678

**Published:** 2020-07-09

**Authors:** Kira Behm, Eszter Fazekas, Martin J. Paterson, Filipe Vilela, Ruaraidh D. McIntosh

**Affiliations:** ^1^ Institute of Chemical Sciences Heriot-Watt University Edinburgh EH14 4AS UK

**Keywords:** amine bis(phenolate), flow chemistry, photosensitiser, singlet oxygen, titanium

## Abstract

A series of novel bimetallic Ti^IV^ amine bis(phenolate) complexes was synthesised and fully characterised. X‐ray crystallography studies revealed distorted octahedral geometries around the Ti centres with single or double oxo‐bridges connecting the two metals. These robust, air‐ and moisture‐stable complexes were employed as photosensitisers generating singlet oxygen following irradiation with visible light (420 nm) LED module in a commercial flow reactor. All five complexes showed high activity in the photo‐oxygenation of α‐terpinene and achieved complete conversion to ascaridole in four hours at ambient temperature. The excellent selectivity of these photosensitisers towards ascaridole (vs. transformation to *p*‐cymene) was demonstrated with control experiments using a traditional TiO_2_ catalyst. Further comparative studies employing the free pro‐ligands as well as a monometallic analogue highlighted the importance of the ‘TiO_2_‐like’ moiety in the polymetallic catalysts. Computational studies were used to determine the nature of the ligand to metal charge transfer (LMCT) states and singlet–triplet gaps for each complex, the calculated trends in the UV‐vis absorption spectra across the series agreed well with the experimental results.

## Graphical abstract

**Bimetallic titanium‐oxo complexes** generate singlet oxygen under visible light irradiation, which is used to photo‐oxygenate α‐terpinene to ascaridole under flow conditions. The activity of five titanium amine bis(phenolate) complexes was investigated and the compounds were found to be selective for the production of ascaridole over *p*‐cymene.

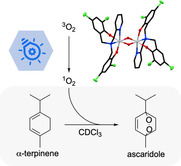

## Introduction

Photocatalytic transformations are increasingly attractive as they offer environmentally friendly pathways for a range of chemical processes including water purification, water splitting, CO_2_ conversion and organic syntheses.^1^, ^2^, ^3^, ^4^ The rapid rise of flow photoreactors, coupled with affordable LED light‐sources in the past decade has further promoted the development of both heterogeneous and homogeneous photocatalytic systems.^5^ Catalysts that display a strong response to visible light (400–700 nm) are desirable, as they are safer to use and enable the efficient utilisation of natural sunlight whilst preventing potential side‐reactions and catalyst degradation that commonly occur under high energy UV irradiation (<400 nm).^2^, ^6^

Various forms of the ubiquitous titania (TiO_2_) catalysts have been successfully applied in classical UV‐activated photochemistry due to their abundance, low toxicity and large band gap energy.^7^ To extend the absorption into the visible region, however, TiO_2_ needs to be doped with additives including metals (Cd, Ce, Mn, Bi etc.) and non‐metallic anions.^8^, ^9^, ^10^ While the incorporation of these (often expensive or toxic) components significantly increases the photocatalytic efficiency, it can simultaneously lower the thermal stability of the catalyst and create undesired electron traps.^10^ Other TiO_2_‐like materials, such as polyoxotitanates (POTs) have also been investigated as photocatalysts.^11^ Modifications of POTs for enhanced visible light harvesting include heterometallic doping or the incorporation of simple functional ligands that influence the absorption.^12^

While heterogeneous systems usually benefit from higher stability and easier separation, the application of homogeneous photocatalysts often allows better fine‐tuning that leads to enhanced activity and selectivity,^13^ moreover they enable convenient, solution‐phase reaction monitoring and mechanistic studies. In the past decades a variety of well‐defined, soluble molecular catalysts have been designed for photochemical processes comprising photo‐organocatalysts (including organic dyes) and metal complexes.^14^, ^15^, ^16^ The latter group is dominated by polypyridyl complexes of Ru and Ir for photoredox reactions,^17^, ^18^, ^19^ while transition metal complexes of porphyrin and phthalocyanine‐derivatives have been frequently applied as photosensitisers.^20^, ^21^

An important photocatalytic reaction is the generation of singlet oxygen (^1^O_2_), which can be employed as a green oxidising agent in dye degradation, wastewater disinfection, cancer treatment and synthetic processes.^22^, ^23^, ^24^ This technique requires a photosensitiser that can exist in a relatively long‐lived triplet excited state, by efficiently undergoing inter‐system crossing following absorption of a photon of the appropriate wavelength.^25^, ^26^ The energy is consequently transferred onto ground‐state triplet oxygen molecules (^3^O_2_) converting them into metastable ^1^O_2_.^27^, ^28^, ^29^ Most commonly, organic dyes such as methylene blue have been applied as photosensitisers.^30^, ^31^ More recently, complexes of precious metals (Ru, Re, Os, Ir and Pt) have also been used, often as bimodal catalysts with large, light‐harvesting moieties incorporated into the ligand framework.^32^, ^33^, ^34^, ^35^, ^36^, ^37^

Despite the popularity of TiO_2_ and POT‐based systems, the utilisation of discrete, well‐defined complexes of Ti is underexplored in the field of photocatalysis.^38^, ^39^, ^40^ Our group has had a longstanding interest in the application of this abundant and non‐toxic metal, particularly in combination with amine bis(phenolate) (ABP) ligands due to their convenient synthesis and easily tailored stereoelectronic properties.^41^, ^42^ The employment of Ti ABP complexes in photocatalysis is promising, as they combine the benefits of TiO_2_‐like motifs (via Ti−O and Ti−O−Ti moieties) with the benefit of adjusting their light absorption into the visible region (as indicated by their yellow‐red colour). Here, we report the first examples of bimetallic homogeneous ‘TiO_2_‐like’ complexes utilised as visible‐light‐activated photosensitisers in the generation of ^1^O_2_.

## Results and Discussion

### Synthesis

**Figure 1:**
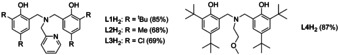
Structures of amine bis(phenol) pro‐ligands (**L1H_2_‐L4H_2_**).

A series of amine bis(phenol) pro‐ligands (**L1H_2_**–**L4H_2_**) was synthesised via double Mannich condensations following modified literature procedures.^43^ Using water as solvent, the desired compounds formed with high yields (69–87 %) as white powders following trituration in methanol (Figure 1).

**Figure 2:**
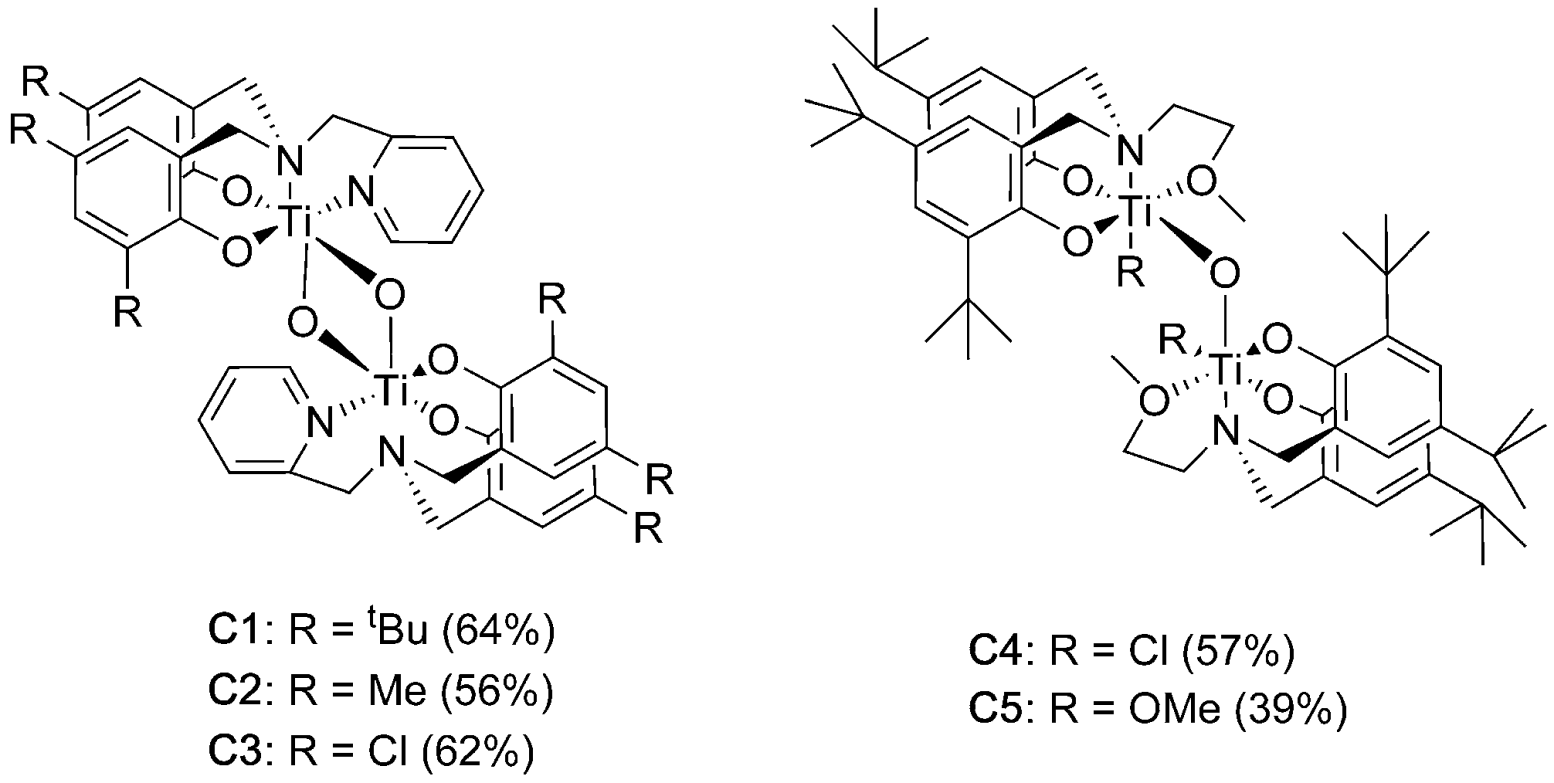
Structures of bimetallic Ti−O−Ti complexes (**C1**–**C5**).

The corresponding Ti^IV^ complexes were formed using two different metal precursors; **C1**, **C2**, **C4** and **C5** were synthesised through the deprotonation of the pro‐ligands with NaH, followed by the addition of TiCl_4_, while **C3** was formed via the direct addition of Ti(O*i*Pr)_4_ at ambient temperature. Coordination of the ligands to the Ti centre was confirmed via the splitting of *N*‐methylene resonances (4.93–2.57 ppm for **C1**) in the ^1^H NMR spectra. The bimetallic nature of the complexes was corroborated using ESI mass spectrometry and single crystal X‐ray crystallography. The compounds were purified via recrystallisation from diethyl ether or chloroform to give complexes **C1–C5** with moderate to high yields (39–64 %) as yellow and red solids (Figure 2).

### Crystallography

Single crystals, suitable for X‐ray diffraction studies, were grown via the slow evaporation of diethyl ether (**C2** and **C3**), chloroform (**C4**) or methanol (**C5**) solutions of the compounds. Using non‐dried crystallisation solvents has reliably led to the formation of bimetallic complexes with Ti−O−Ti oxo‐bridges. In **C5**, the propoxide groups on the Ti centres were displaced with methoxy ligands originating from the methanol solvent. Interestingly, for ligands **L1**–**L3**, double oxo‐bridged bimetallic complexes (**C1**–**C3**) were formed, whereas for ligand **L4**, only single oxo‐bridged complexes **C4** and **C5** were obtained. A range of procedures were explored in an attempt to form equivalent complexes with all ligands, however, the difference in the number of oxo‐bridges persisted, regardless of the synthetic method or the crystallisation solvent used.

**Figure 3:**
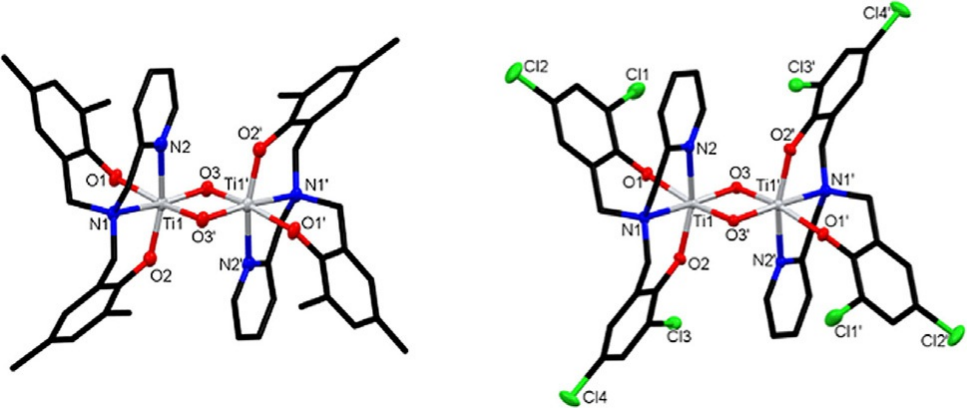
Molecular structures of **C2** (left) and **C3** (right) with ellipsoids set at the 50 *%* probability level. Hydrogen atoms have been omitted for clarity. Selected bond lengths for **C2** (Å): Ti1‐O1 1.9249(11), Ti1‐O2 1.8827(13), Ti1‐O3 1.7748(12), Ti1‐O3*’* 1.9550(11), Ti1‐N1 2.3261(14), Ti1‐N2 2.2280(15). Selected bond angles for **C2** (°): O1‐Ti1‐N1 83.84(5), O1‐Ti1‐O3 105.35(5), O3‐Ti1‐O3*’* 83.54(5), N1‐Ti1‐N2 73.31(5), N2‐Ti1‐O2 160.71(5). Selected bond lengths for **C3** (Å): Ti1‐O1 1.9332(12), Ti1‐O2 1.8965(13), Ti1‐O3 1.7743(12), Ti1‐O3*’* 1.9495(12), Ti1‐N1 2.3169(14), Ti1‐N2 2.2063(15). Selected bond angles for **C3** (°): O1‐Ti1‐N1 85.63(5), O1‐Ti1‐O3 103.23(5), O3‐Ti1‐O3*’* 83.48(5), N1‐Ti1‐N2 73.82(5), N2‐Ti1‐O2 158.47(5).

**Figure 4:**
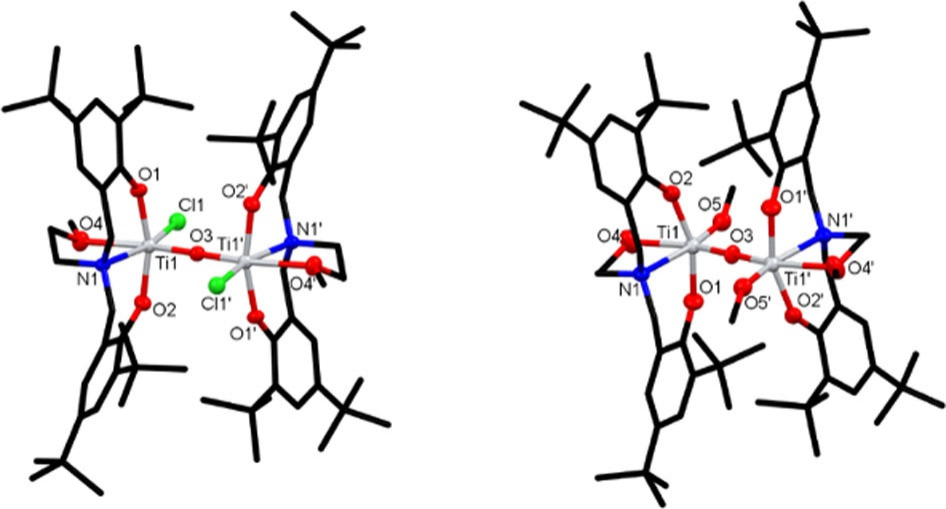
Molecular structures of **C4** (left) and **C5** (right) with ellipsoids set at the 50 *%* probability level. Hydrogen atoms have been omitted for clarity. Selected bond lengths for **C4** (Å): Ti1‐O1 1.8555(15), Ti1‐O2 1.8543(15), Ti1‐O3 1.8002(4), Ti1‐O4 2.3046(16), Ti1‐N1 2.2671(18), Ti1‐Cl1 2.3312(6). Selected bond angles for **C4** (°): O1‐Ti1‐N1 83.36(7), O1‐Ti1‐O3 95.67(5), O3‐Ti1‐Cl1 101.20(2), O1‐Ti1‐O2 163.69(7), O1‐Ti1‐Cl1 93.68(5). Selected bond lengths for **C5** (Å): Ti1‐O1 1.884(4), Ti1‐O2 1.889(4), Ti1‐O3 1.8066(11), Ti1‐O4 2.305(4), Ti1‐O5 1.807(4), Ti1‐N1 2.288(5). Selected bond angles for **C5** (°): O1‐Ti1‐N1 83.26(18), O1‐Ti1‐O3 96.31(14), O3‐Ti1‐O5 104.96(14), O1‐Ti1‐O2 161.29(19), O1‐Ti1‐O5 94.79(19).

All complexes exhibit a distorted octahedral geometry around the metal centres, with N2‐Ti‐O2 (**C2** and **C3**) and O1‐Ti‐O2 (**C4** and **C5**) bond angles ranging from 158.47(5)° to 163.69(7)°. Notably, the phenolate groups take up a *cis* arrangement, when ligands with methylpyridine side‐arms were used (**L1**–**L3**, Figure 3), whereas using **L4**, the decreased steric bulk of the methoxyethyl side‐arm favours the phenolate groups arranged in a *trans* position (Figure 4). Similar behaviour was observed in related Ti complexes investigated by Mountford and co‐workers.^43^ The metal‐ligand bond lengths in the double‐bridged complexes **C2** and **C3** are slightly longer than in complexes **C4** and **C5**, which could be attributed to the extra steric bulk of the methylpyridine side‐arm and the presence of the sterically strained four‐membered ring. For example, in **C2**, the Ti–phenolate bond lengths are 1.9249(1) Å and 1.8827(1) Å, whereas in **C4**, the corresponding values are 1.855(2) Å and 1.854(2) Å, respectively. Overall, the bond lengths and bond angles in all four novel bimetallic complexes are comparable to related Ti ABP derivatives reported in the literature.^44^, ^45^, ^46^

### UV‐vis spectroscopy studies

**Figure 5:**
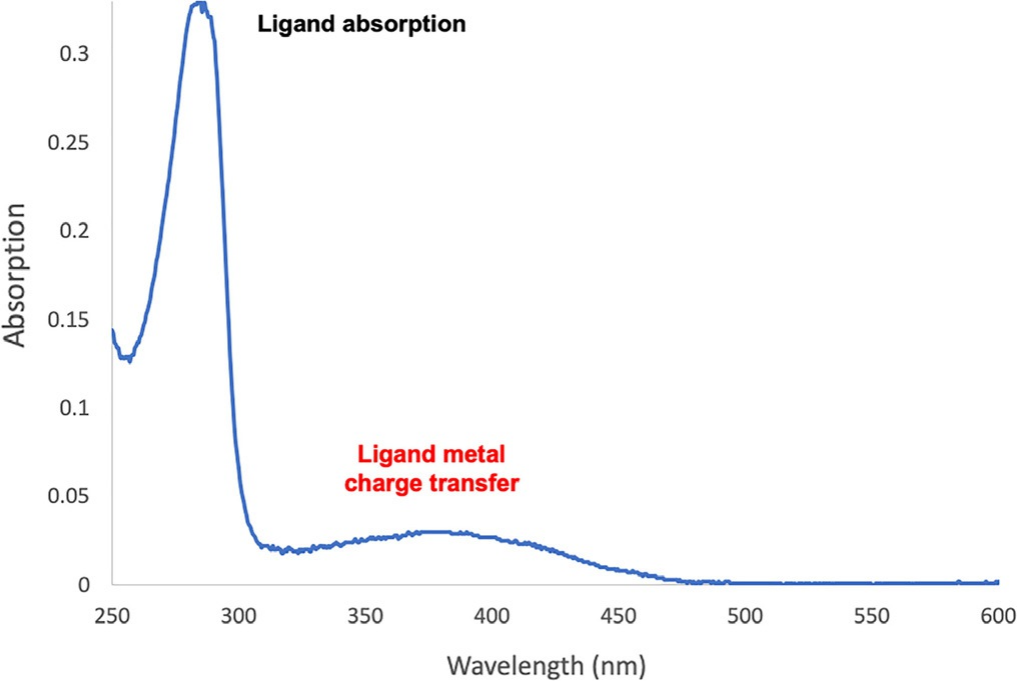
UV‐vis spectrum of **C4** showing the ligand and complex absorption maxima.

Solution phase UV‐vis spectra of complexes **C1**–**C5** were recorded using chloroform as the solvent in order to determine the absorption profiles of the compounds. The spectra of complexes (e.g. **C4**, Figure 5) show a larger absorption band at 280 nm, corresponding to the absorption maximum of the ligand, which was confirmed with control experiments (Figure S18). A second, weaker absorption band was observed at 378 nm, which corresponds to a ligand metal charge transfer (LMCT). All dimeric Ti complexes exhibited a similar absorption maximum (332–378 nm, Table 1), with significant response detectable in the visible region (>400 nm), which was also indicated by their yellow to red colour. Importantly, this property allowed the photosensitisation experiments to be carried out with irradiation at the wavelength of 420 nm, which offers an advantage over traditional TiO_2_ catalysts, such as anatase, that does not absorb in the visible region.

**Table 1:** UV‐vis absorption maxima of complexes **C1**–**C5**.

	Complex	*λ*_max_ (nm)^[a]^	
	**C1**	334	
	**C2**	334	
	**C3**	332	
	**C4**	378	
	**C5**	338

[a] Spectra were recorded with 0.0003 m solution of the complexes in CHCl_3_ at ambient temperature.

### Photocatalytic activity

**Scheme 1:**
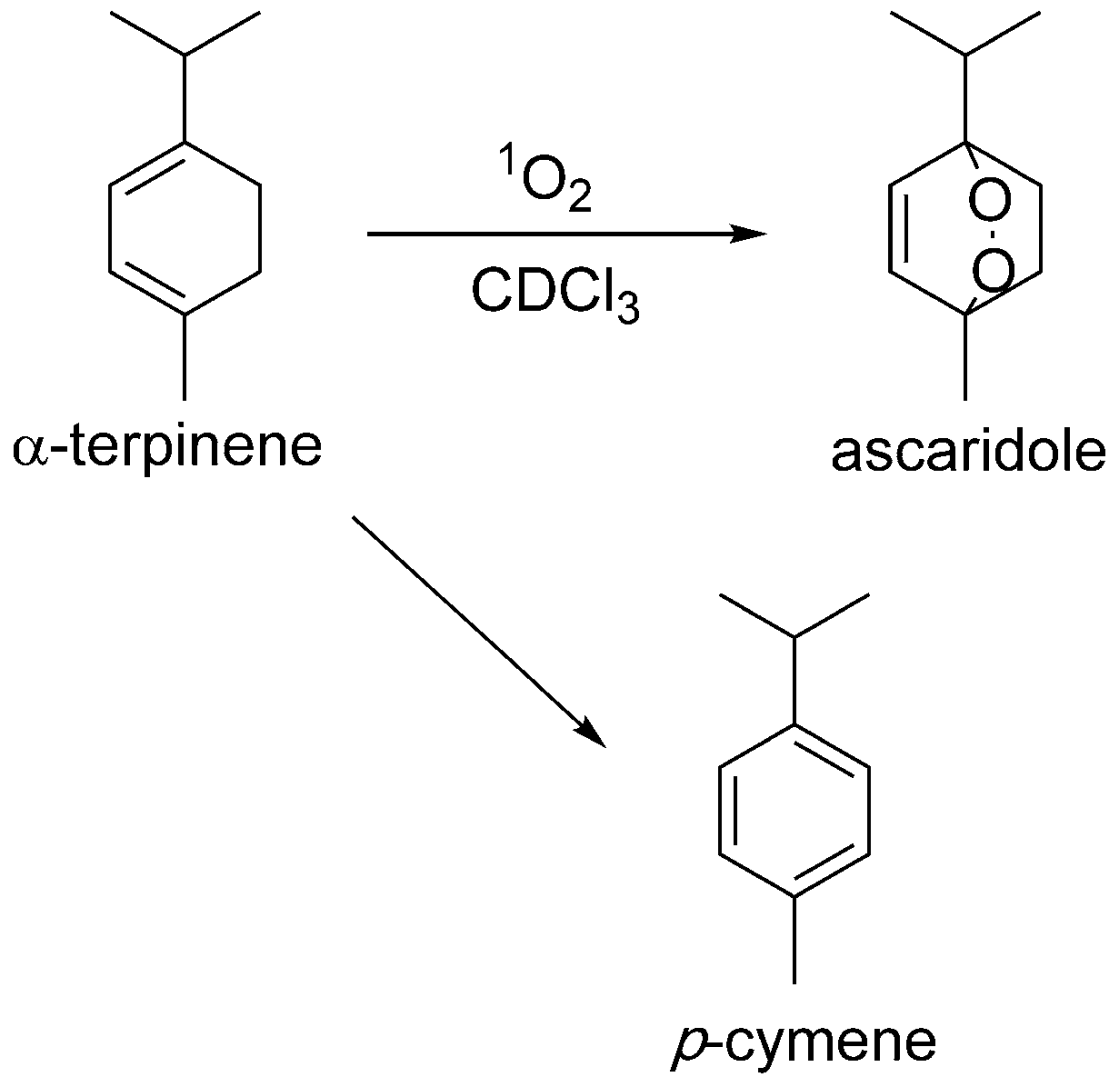
Synthesis of ascaridole from α‐terpinene and competing side reaction to form *p*‐cymene.

Complexes **C1**–**C5** were screened for activity as triplet photosensitisers in the generation of singlet oxygen using a commercial flow photoreactor under visible light (420 nm) irradiation. The activity was monitored via the photo‐oxygenation reaction of α‐terpinene towards ascaridole, which only proceeds in the presence of ^1^O_2_ (Scheme 1).^47^ All experiments were carried out in CDCl_3_ solvent as deuterated solvents are known to be beneficial for increased ^1^O_2_ lifetime and enable convenient sampling for the determination of conversion via ^1^H NMR spectroscopy (Figure S20).^27^, ^48^ The reaction mixtures were saturated with O_2_ prior to the experiments and flow rates of both the substrate solution and a continuous oxygen supply were kept constant at 1 mL min^−1^. A schematic of the flow reactor setup can be found in the supporting information (Figure S19).

**Figure 6:**
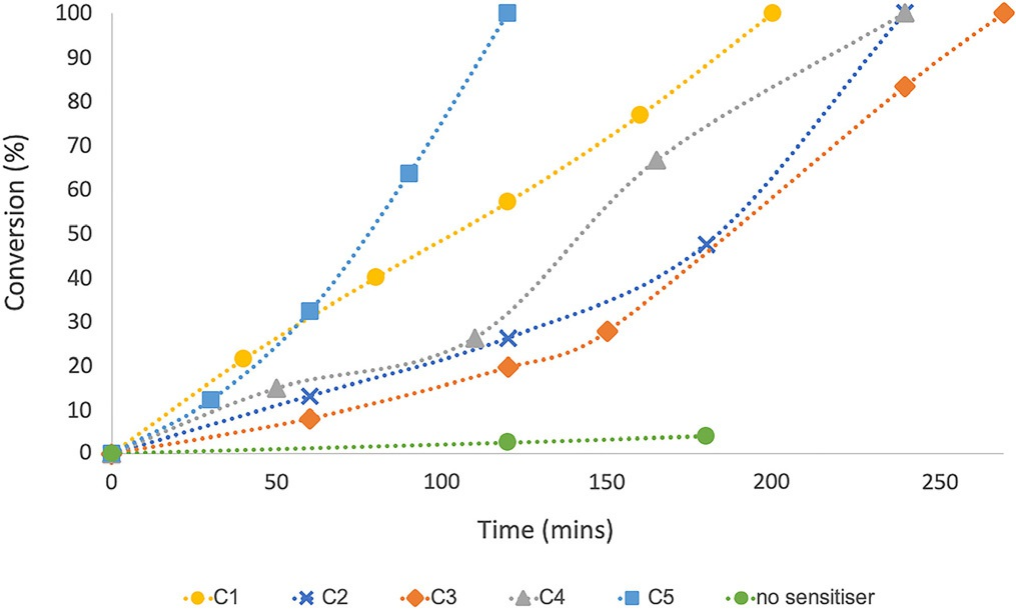
Conversion of α‐terpinene to ascaridole vs. time.

Using 5 mol % catalyst loading, all five complexes showed good activity in the production of ^1^O_2_ at ambient temperature (Figure 6). α‐Terpinene was selectively converted to ascaridole, the formation of common by‐products such as *p*‐cymene was not detected in the reaction mixtures.^49^ On the other hand, comparative studies using TiO_2_ (P25) under identical reaction conditions afforded exclusively transformation into *p*‐cymene as indicated by the appearance of an aromatic resonance at 7.11 ppm in the ^1^H NMR spectra of reaction mixtures. The lack of oxygenated products in these experiments confirms that traditional TiO_2_ catalysts are not suitable for ^1^O_2_ generation under visible light irradiation. Notably, control experiments in the absence of photosensitisers achieved a low conversion of 4 % in three hours, which can be attributed to the commonly observed self‐sensitisation phenomenon.^34^ These results highlight that the presence of the Ti complexes was essential for efficient ^1^O_2_ generation.

**Figure 7:**
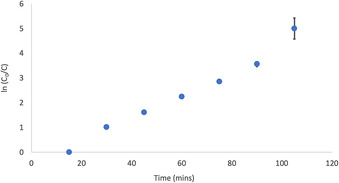
α‐Terpinene ln(C_0_/C) vs. time using complex **C5**.

The most active photosensitiser was complex **C5**, achieving full conversion in two hours (Figure 7). Kinetic studies with samples taken at 15 minute intervals showed a near linear reaction profile, which revealed a pseudo first order dependence on substrate concentration, comparable kinetic profiles were previously observed in the photo‐oxygenation of α‐terpinene.^50^, ^51^ Similar rates were observed using **C1**, which reached full conversion of the α‐terpinene in three hours. While these reaction rates generally fall below those achieved with precious metal (Re and Ir) photosensitisers,^32^, ^52^, ^53^ it is clearly demonstrated that these simple and stable Ti ABP complexes are capable to reach ^1^O_2_ generation activity levels comparable to TiO_2_ and POT‐based systems.

Complexes **C2**, **C3** and **C4** exhibit a slower rate in the first 30 minutes of the reaction, therefore full conversion is reached in up to four hours. This lag period suggested an initial transformation of these photosensitisers into a different, catalytically species. However, the photostability of **C4** was investigated under 420 nm irradiation for 24 hours and the complex remained unchanged according to ^1^H NMR spectroscopy studies. Furthermore, mass spectrometry analysis of the reaction mixtures after the photocatalytic experiment verified the presence of the initial bimetallic complexes. It was therefore concluded that the active photosensitisers were the dimeric Ti−O−Ti bridged structures. It appears that there is an acceleration in reaction rate at higher conversions, which can be attributed to a combination of different effects, such as some autocatalytic behaviour of the ascaridole formed, or the increased ratio of ^1^O_2_ versus α‐terpinene.

With respect to structural properties, *tert*‐butyl groups on the ligands showed a positive impact on the production of ^1^O_2_, likely due to the increased solubility of these complexes (**C1**, **C4**, **C5**).

**Figure 8:**
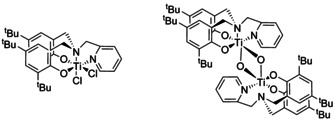
Structures of monometallic vs. bimetallic Ti complexes of ligand **L1H_2_**.

Importantly, it was shown that the presence of the Ti−O−Ti moiety is crucial for the efficient photosensitisation: Control reactions with a monometallic complex (**L1**)TiCl_2_ (Figure 8) only achieved 18 % conversion in four hours *cf*. 100 % was achieved in three hours with the bimetallic μ‐oxo‐bridged analogue **C1**. Moreover, the remarkable conversion achieved with **C5** showed, that the activity is enhanced by the methoxy groups on the metal centres, which further extend the oxo‐bridged framework. Control experiments revealed that the free pro‐ligands (**L1H_2_**, **L3H_2_** and **L4H_2_**) also exhibit some catalytic activity under these reaction conditions, however, they yielded similarly low conversions (9–28 % after 4 h).

**Figure 9:**
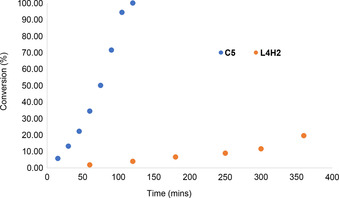
α‐Terpinene conversion in the presence of **C5** vs. **L4H_2_**.

Figure 9 compares the activities of **C5** and the corresponding pro‐ligand **L4H_2_**. Experiments without oxygen bubbling showed that it is essential to have a constant supply of O_2_ to achieve high conversions.

### Computational studies

Each of the target molecules (**C2**–**C5**) was optimised using CAM‐B3LYP/6–311G(d,p) starting from the crystal structure coordinates. A polarizable continuum model (PCM) was used to model a chloroform solvent (dielectric constant=4.7113). Time‐dependent (TD) CAM‐B3LYP was used to determine the excited singlet and triplet electronic states. This has previously been shown to compare well to high‐order correlated wavefunction response approaches,^54^ and perform well in describing neutral and charged TiO_2_ clusters.^55^ S_0_ and T_1_ states were optimised and the analytical Hessian was confirmed as positive definite to verify the structures as minima. The nature of the excited electronic states was determined using the response eigenvectors with the canonical Kohn–Sham orbitals. All computations were performed using a local version of Gaussian16.^56^

**Figure 10:**
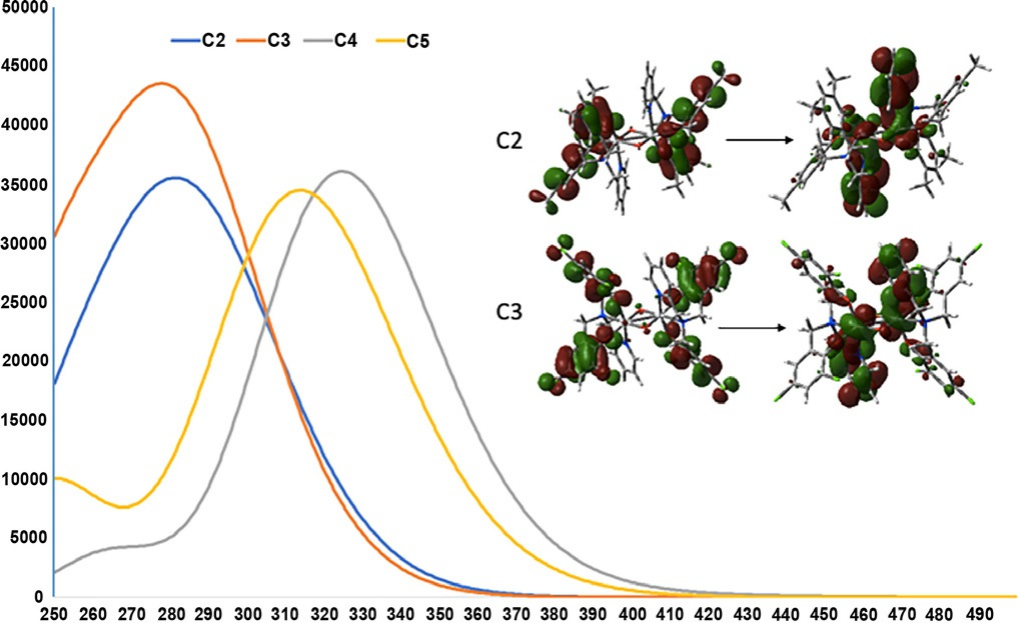
(TD)‐CAM‐B3LYP calculated absorption spectra for target molecules **C2–C5**. Inset are dominant particle‐hole transitions for **C2** and **C3** highlighting the nature of the bright electronic states for each species through LMCT *π* to dπ* transitions. Spectra generated by convoluting a Gaussian (FWHM=20 nm) over lowest 30 singlet states.

All optimised geometries agree well with the crystal structures. The calculated absorption spectra agree reasonably well with the experimental results for clusters of this size (with a general blue‐shift). All excited states are very mixed with many LMCT transitions contributing to each state. The LMCT transitions are from π orbitals, localised on the aromatic units, to orbitals of Ti localised d‐character mixed with higher π* orbitals (Figure 10).

The dominant particle–hole pair is shown in Table 2 (for each molecule this is around 40–50 % of the state). There is a large density of low‐lying electronic states and for all molecules the lowest excited state is generally a quasi‐degenerate pair of one bright and one dark state (Table 2). For **C4** and **C5** the brightest state is not this lowest pair but a (slightly) higher lying one. Each molecule **C2–C4** has a low‐lying triplet state that is well separated from all other excited electronic states. All molecules show a similar energy gap from this triplet to the ground state singlet. For each molecule the T_1_ state has a significant component of HOMO–LUMO particle‐hole character, and like the singlet manifold these involve LMCT (π/dπ*) character. Consistent with this the triplet states have elongated Ti−O bonds relative to the ground state structures but generally a similar topology.

**Table 2:** (TD)‐CAM‐B3LYP calculated optical properties for target molecules.^[a]^

	State	Δ*E* ^vert^ [eV]	Character	p–h	osc. strength (f)	Δ*E*_S/T_ [eV]
**C2**	S_1_/S_2_	4.14	LMCT π–dπ*	H‐1→L	0.3390	2.36
**C3**	S_1_/S_2_	4.23	LMCT π–dπ*	H‐1→L	0.4336	2.39
**C4**	S_1_/S_2_	3.17	LMCT π–dπ*	H→L	0.0021	2.38
	S_10_	3.87	LMCT π–dπ*	H‐1→L	0.5332	–
**C5**	S_1_/S_2_	3.82	LMCT π–dπ*	H‐1→L	0.1961	2.41
	S_3_	3.98	LMCT π–dπ*	H→L	0.5116	–

[a] Vertical excitation energies to lowest and the brightest state in the spectral range up to 250 nm, associated oscillator strengths, character, particle–hole (p–h) nature relative to HOMO and LUMO, and (radiative) singlet–triplet energy gaps from lowest triplet T_1_ state.

## Conclusions

In conclusion, a series of five novel bimetallic Ti−O−Ti bridged amine bis(phenolate) complexes were synthesised and employed as photosensitisers in the generation of ^1^O_2_. Unlike their heterogeneous TiO_2_‐based counterparts, these soluble and well‐defined aggregates showed significant response to visible light, which allowed them to be screened as photosensitisers in a commercial flow reactor under 420 nm LED irradiation. All complexes have efficiently converted α‐terpinene to ascaridole between two to four hours of residence time, moreover excellent selectivity (>99 %) towards ascaridole (vs. *p*‐cymene) was achieved as confirmed via control experiments using TiO_2_. Comparative studies with the free pro‐ligand and a monometallic analogue showed that the presence of the ‘TiO_2_‐like’ Ti−O−Ti moiety is crucial to achieve efficient photo‐oxygenation. The LMCT states and singlet–triplet gaps for each complex were investigated using computational methods, the calculated UV‐vis absorption spectra agreed reasonably well with the experimental observations. These results highlight that smaller, modular complexes of Ti^IV^ can be competitive alternatives of the widely researched TiO_2_ and POT photocatalyst systems. Moreover, fine‐tuning of the ligand design may extend the absorption further into the visible region, which would allow the utilisation of this inexpensive and non‐toxic metal to replace Ru‐ and Ir‐based catalysts in a wider scope of photochemical reactions. The apparent interplay between activity and ligand substituents/bridges between the Ti centres will be further investigated along with the breadth of reactivity in photocatalysis that these complexes can display.

## Experimental Section

**General considerations**: Starting materials were purchased and used as received from Merck, Acros and Fluorochem. Unless stated otherwise, experiments were carried out under ambient atmosphere. Dry solvents were purified in an MBRAUN SPS‐800 and stored over activated 4 Å molecular sieves under a dry N_2_ atmosphere. NMR spectroscopy data was acquired with a Bruker AVIII 300 MHz instrument or Bruker AVIII 400 MHz instrument at 298 K in CDCl_3_. Electrospray ionisation mass spectrometry (ESI) was recorded using a Bruker micrOTOF II. Solution‐state UV/Vis spectra were recorded on a Shimadzu UV‐2550 system with 10 mm quartz cuvettes.

**General procedure for the synthesis of pro‐ligands L1H_2_–L4H_2_**: Pro‐ligands L1H_2_–L4H_2_ were reported previously and were prepared using an adapted literature procedure.^57^, ^58^, ^59^, ^60^

Phenol (2,4‐di‐*tert*‐butylphenol, 2,4‐dimethylphenol or 2,4‐dichlorophenol, 24.5 mmol) was suspended in water (50 mL). 2‐picolylamine or 2‐methoxyethylamine (12.3 mmol) and aqueous formaldehyde solution (37 % wt., 2.0 mL, 25 mmol) were added and the mixture was heated at reflux for 48 hours. The suspension was filtered and the resulting off‐white solid was triturated in methanol to yield the pro‐ligands as a white powder.

**Data for L1H_2_**: (5.82 g, 85 %). ^1^H NMR (300 MHz, CDCl_3_): *δ* 10.54 (s, 2 H, O*H*), 8.69 (m, 1 H, Ar*H*), 7.69 (td, *J=*7.7, 1.8 Hz, 1 H, Ar*H*), 7.28 (m, 1 H, Ar*H*), 7.23 (d, *J=*2.5 Hz, 2 H, Ar*H*), 7.12 (d, *J=*7.8 Hz, 1 H, Ar*H*), 6.94 (d, *J=*2.5 Hz, 2 H, Ar*H*), 3.85 (s, 2 H, Py‐C*H*_2_), 3.81 (s, 4 H, Ar‐C*H*_2_), 1.41 (s, 18 H, CC*H*_3_), 1.29 (s, 18 H, CC*H*_3_).

**Data for L2H_2_**: (3.33 g, 68 %). ^1^H NMR (300 MHz, CDCl_3_): *δ* 10.44 (s, 2 H, O*H*), 8.71 (m, 1 H, Ar*H*), 7.70 (td, *J=*7.7, 1.8 Hz, 1 H, Ar*H*), 7.28 (m, 1 H, Ar*H*), 7.12 (d, *J=*7.8 Hz, 1 H, Ar*H*), 6.87 (s, 2 H, Ar*H*), 6.71 (s, 2 H, Ar*H*), 3.84 (s, 2 H, Py‐C*H*_2_), 3.75 (s, 4 H, Ar‐C*H*_2_), 2.22 (s, 12 H, C*H*_3_).

**Data for L3H_2_**: (3.88 g, 69 %). ^1^H NMR (300 MHz, CDCl_3_): *δ* 11.36 (s, 2 H, O*H*), 8.72 (m, 1 H, Ar*H*), 7.80 (td, *J=*7.7, 1.8 Hz, 1 H, Ar*H*), 7.36 (m, 1 H, Ar*H*), 7.30 (d, *J=*2.5 Hz, 2 H, Ar*H*), 7.19 (d, *J=*7.9 Hz, 1 H, Ar*H*), 6.97 (d, *J=*2.5 Hz, 2 H, Ar*H*), 3.87 (s, 2 H, Py‐C*H*_2_), 3.81 (s, 4 H, Ar‐C*H*_2_).

**Data for L4H_2_**: (5.39 g, 87 %). ^1^H NMR (300 MHz, CDCl_3_): *δ* 8.48 (bs, 2 H, O*H*), 7.21 (d, *J=*2.5 Hz, 2 H, Ar*H*), 6.88 (d, *J=*2.4 Hz, 2 H, Ar*H*), 3.74 (s, 4 H, Ar‐C*H*_2_), 3.56 (t, *J=*5.2 Hz, 2 H, C*H*_2_), 3.47 (s, 3 H, OC*H*_3_), 2.74 (t, *J=*5.2 Hz,2 H, C*H*_2_), 1.41 (s, 18 H, CC*H*_3_), 1.27 (s, 18 H, CC*H*_3_).

**General procedure for the synthesis of amine bis(phenolate) Ti−O−Ti complexes (C1–C5)**: C1, C2 and C4: Pro‐ligands L1H_2_, L2H_2_ and L4H_2_ (1.0 mmol) were dissolved in dry toluene (30 mL). Sodium hydride (60 % wt. dispersion in mineral oil, 0.08 g, 2.0 mmol) was added and the solution was stirred for 30 minutes. TiCl_4_ (1 m solution in toluene, 1.0 mL, 1.0 mmol) was added slowly. The mixture was stirred for three hours, the solvent was removed under reduced pressure and the crude product was dissolved in methanol (50 mL). Sodium methoxide (0.11 g, 2.0 mmol) was added and the solution was stirred for four hours. The crude product was washed with deionised water and recrystallised from diethyl ether. (The addition of sodium methoxide was omitted for C4 and the crude product was recrystallised from chloroform).

**C3 and C5**: Pro‐ligands L3H_2_ and L4H_2_ (1.0 mmol) were dissolved in THF (20 mL). Ti(O*i*Pr)_4_ (0.28 g, 0.30 mL, 1.0 mmol) was added and the solution was stirred for two hours. The solvent was removed under reduced pressure and the crude product was recrystallised from diethyl ether or methanol.  Deposition Numbers 1992707 (C2), 1992708 (C3), 1992709 (C4), 1992710 (C5) contain the supplementary crystallographic data for this paper. These data are provided free of charge by the joint Cambridge Crystallographic Data Centre and Fachinformationszentrum Karlsruhe Access Structures service.

**Data for (L1)TiCl_2_**: (L1)TiCl_2_ (0.44 g, 69 %) was prepared according to literature procedures^43^ for comparison of photosensitising properties. ^1^H NMR (400 MHz, CDCl_3_): *δ* 9.04 (m, 1 H, Ar*H*), 7.52 (td, *J=*7.7, 1.6 Hz, 1 H, Ar*H*), 7.29 (m, 1 H, Ar*H*), 7.05 (d, *J=*2.2 Hz, 1 H, Ar*H*), 6.90 (d, *J=*2.2 Hz, 1 H, Ar*H*), 6.79 (d, *J=*2.3 Hz, 1 H, Ar*H*), 5.39 (d, *J=*13.1 Hz, 1 H, Py‐C*H*_2_), 5.01 (d, *J=*14.8 Hz, 1 H, Py‐C*H*_2_), 3.91 (m, 2 H, Ar‐C*H*_2_), 3.55 (d, *J=*13.1 Hz, 1 H, Ar‐C*H*_2_), 3.08 (d, *J=*13.0 Hz, 1 H, Ar‐C*H*_2_), 1.57 (s, 8 H, CC*H*_3_), 1.42 (s, 2 H, CC*H*_3_), 1.33 (s, 8 H, CC*H*_3_), 1.30 (s, 8 H, CC*H*_3_), 1.27 (s, 2 H, CC*H*_3_), 1.11 (s, 8 H, CC*H*_3_).

**Data for C1**: (0.39 g, 64 %). ^1^H NMR (400 MHz, CDCl_3_): *δ* 8.65 (m, 2 H, Ar*H*), 7.17 (t, *J=*7.7 Hz, 2 H, Ar*H*), 7.02 (d, *J=*2.0 Hz, 2 H, Ar*H*), 6.88 (d, *J=*2.0 Hz, 2 H, Ar*H*), 6.84 (s, 1 H, Ar*H*), 6.83 (s, 1 H, Ar*H*), 6.67 (m, 4 H, Ar*H*), 6.46 (d, *J=*7.7 Hz, 2 H, Ar*H*), 4.93 (d, *J=*13.7 Hz, 2 H, C*H*_2_), 4.75 (d, *J=*12.6 Hz, 2 H, C*H*_2_), 3.50 (m, 4 H, C*H*_2_), 3.16 (d, *J=*11.8 Hz, 2 H, C*H*_2_), 2.57 (d, *J=*13.0 Hz, 2 H, C*H*_2_), 1.31 (s, 18 H, CC*H*_3_), 1.24 (s, 18 H, CC*H*_3_), 1.06 (s, 18 H, CC*H*_3_), 0.90 (s, 18 H, CC*H*_3_). ^13^C NMR (75.5 MHz, CD_2_Cl_2_): *δ* 161.2 (*C*), 159.1 (*C*), 156.7 (*C*), 151.7 (*C*H), 139.8 (*C*), 138.5 (*C*), 137.9 (*C*H), 136.2 (*C*), 134.4 (*C*), 126.1 (*C*), 125.8 (*C*), 124.9 (*C*H), 124.1 (*C*H), 123.7 (*C*H), 122.8 (*C*H), 122.5 (*C*H), 121.5 (*C*H), 64.1 (*C*H_2_), 62.4 (*C*H_2_), 59.3 (*C*H_2_), 35.2 (*C*CH_3_), 35.0 (*C*CH_3_), 34.3 (*C*CH_3_), 34.0 (*C*CH_3_), 31.9 (*C*H_3_), 31.8 (*C*H_3_), 30.4 (*C*H_3_), 30.3 (*C*H_3_). HRMS (ESI): *m*/*z* [*M*+H]^+^ 1213.6652; calcd [*M*+H]^+^ 1213.6675.

**Data for C2**: (0.24 g, 56 %). ^1^H NMR (400 MHz, CDCl_3_): *δ* 8.66 (m, 2 H, Ar*H*), 7.36 (t, *J=*7.6 Hz, 2 H, Ar*H*), 6.73 (m, 8 H), 6.37 (d, *J=*10.0 Hz, 4 H, Ar*H*), 4.73 (d, *J=*12.6 Hz, 2 H, C*H*_2_), 4.63 (d, *J=*14.0 Hz, 2 H, C*H*_2_), 3.78 (d, *J=*12.9 Hz, 2 H, C*H*_2_), 3.55 (d, *J=*15.0 Hz, 2 H, C*H*_2_), 3.26 (d, *J=*12.1 Hz, 2 H, C*H*_2_), 2.66 (d, *J=*12.6 Hz, 2 H, C*H*_2_), 2.23 (s, 6 H), 1.97 (s, 6 H) 1.90 (s, 6 H), 1.52 (s, 6 H). Due to low solubility in conventional NMR solvents, a ^13^C NMR spectrum with appropriate quality could not be recorded. HRMS (ESI): *m*/*z* [*M*+H]^+^ 877.2903; calcd [*M*+H]^+^ 877.2919.

**Data for C3**: (0.36 g, 62 %). ^1^H NMR (300 MHz, CDCl_3_): *δ* 8.57 (m, 2 H, Ar*H*), 7.57 (td, *J=*7.9, 1.6 Hz, 2 H, Ar*H*), 7.19 (d, *J=*2.7 Hz, 2 H, Ar*H*), 7.01 (d, *J=*2.5 Hz, 2 H, Ar*H*), 6.98 (s, 1 H, Ar*H*), 6.95 (s, 1 H, Ar*H*), 6.89 (m, 2 H, Ar*H*), 6.82 (d, *J=*2.4 Hz, 2 H, Ar*H*), 6.68 (d, *J=*2.5 Hz, 2 H, Ar*H*), 4.73 (d, *J=*15.6 Hz, 2 H, C*H*_2_), 4.66 (d, *J=*13.0 Hz, 2 H, C*H*_2_), 3.74 (d, *J=*11.7 Hz, 2 H, C*H*_2_), 3.63 (d, *J=*15.1 Hz, 2 H, C*H*_2_), 3.33 (d, *J=*13.5 Hz, 2 H, C*H*_2_), 2.82 (d, *J=*12.8 Hz, 2 H, C*H*_2_). Due to solubility issues, a ^13^C NMR spectrum could not be recorded. HRMS (ESI): *m*/*z* [*M*+H]^+^ 1036.8566; calcd [*M*+H]^+^ 1036.8549.

**Data for C4**: (0.35 g, 57 %). ^1^H NMR (300 MHz, CDCl_3_): *δ* 7.09 (d, *J=*2.3 Hz, 4 H, Ar*H*), 6.91 (d, *J=*2.2 Hz, 4 H, Ar*H*), 5.44 (d, *J=*14.0 Hz, 4 H, C*H*_2_), 3.48 (s, 2 H, C*H*_2_), 3.44 (s, 2 H, C*H*_2_), 3.42 (s, 6 H, OC*H*_3_), 3.19 (t, *J=*5.5 Hz, 4 H, C*H*_2_), 2.86 (t, *J=*5.4 Hz, 4 H, C*H*_2_), 1.27 (s, 36 H, CC*H*_3_), 1.05 (s, 36 H, CC*H*_3_). ^13^C NMR (75.5 MHz, CDCl_3_): *δ* 159.7 (*C*), 159.5 (*C*), 140.7 (*C*), 136.4 (*C*), 136.2 (*C*), 134.8 (*C*), 124.6 (*C*), 124.3 (*C*), 124.1 (*C*H), 123.8 (*C*H), 123.7 (*C*H), 123.2 (*C*H), 66.2 (*C*H_2_), 65.9 (*C*H_2_), 63.6 (*C*H_2_), 58.3 (*C*H_2_), 35.4 (*C*CH_3_), 35.2 (*C*CH_3_), 34.6 (*C*CH_3_), 34.4 (*C*CH_3_), 31.8 (O*C*H_3_), 30.3 (*C*H_3_), 30.1 (*C*H_3_), 29.9 (*C*H_3_), 29.2 (*C*H_3_). MS (ESI): *m*/*z* [C_33_H_50_ClNO_4_Ti]^+.^ 608.2923 (monometallic oxo radical fragment).

**Data for C5**: (0.22 g, 39 %). ^1^H NMR (300 MHz, CDCl_3_): *δ* 7.16 (d, *J=*2.4 Hz, 4 H, Ar*H*), 6.71 (d, *J=*2.4 Hz, 4 H, Ar*H*), 4.91 (d, *J=*13.2 Hz, 4 H, C*H*_2_), 3.98 (s, 6 H, OC*H*_3_), 3.28 (s, 6 H, OC*H*_3_), 3.03 (s, 2 H, C*H*_2_), 2.99 (s, 2 H, C*H*_2_), 2.86 (t, *J=*5.8 Hz, 4 H, C*H*_2_), 2.57 (t, *J=*5.8 Hz, 4 H, C*H*_2_), 1.53 (s, 6 H, CC*H*_3_), 1.48 (s, 30 H, CC*H*_3_), 1.29 (s, 6 H, CC*H*_3_), 1.24 (s, 30 H, CC*H*_3_). ^13^C NMR (100.6 MHz, CDCl_3_): *δ* 160.3 (*C*), 159.8 (*C*), 139.0 (*C*), 138.5 (*C*), 135.8 (*C*), 134.9 (*C*), 124.7 (*C*), 124.3 (*C*), 124.2 (*C*H), 123.5 (*C*H), 123.0 (*C*H), 71.9 (*C*H_2_), 64.3 (*C*H_2_), 63.2 (*C*H_2_), 62.0 (*C*H_2_), 35.4 (*C*CH_3_), 35.1 (*C*CH_3_), 34.2 (*C*CH_3_), 34.1 (*C*CH_3_), 32.0 (O*C*H_3_), 31.9 (O*C*H_3_), 30.7 (*C*H_3_), 30.2 (*C*H_3_), 30.1 (*C*H_3_), 30.0 (*C*H_3_). HRMS (ESI): *m*/*z* [*M*+H]^+^ 1193.7087; calcd [*M*+H]^+^ 1193.7087.

**General procedure of** ^**1**^ **O_2_ experiments under flow conditions**: Photosensitised reactions under flow conditions were carried out using a commercial photochemical flow reactor equipped with an LED array emitting a wavelength at 420 nm with 61 W light output and a light intensity of 10.2 W cm^−2^ (easy‐Photochem, Vapourtec Ltd.). A schematic of the flow experiment setup can be found in the supporting information (Figure S19). Titanium complex (C1–C5) (12 mg, 5 mol %) and α‐terpinene (0.16–0.26 mmol) were dissolved in CDCl_3_ (12 mL). The solution was saturated with O_2_ for ten minutes. The solution was then pumped through the photochemical reactor at 1 mL min^−1^. O_2_ was pumped through a second pump at the same flow rate, mixing with the solution at a T‐junction before entering the photochemical reactor. Samples (450 μL) were taken at regular intervals.

**Crystallography**: Single‐crystal X‐ray diffraction data were collected on a Rigaku Oxford diffraction SuperNova diffractometer or a Bruker venture d8 diffractometer with CCD detector with Mo‐Kα radiation (*λ*=0.7107 Å) or Cu‐Kα radiation (*λ*=1.5418 Å). The structures were solved by direct methods using SHELXS or SHELXT and refined by full‐matrix least squares on F^2^ using SHELXL interfaced through Olex2.^61^, ^62^ Molecular graphics for all structures were generated using Mercury.

## Conflict of interest

The authors declare no conflict of interest.

## Supporting information

As a service to our authors and readers, this journal provides supporting information supplied by the authors. Such materials are peer reviewed and may be re‐organized for online delivery, but are not copy‐edited or typeset. Technical support issues arising from supporting information (other than missing files) should be addressed to the authors.

Supplementary
