# Supplementary material for: Discrete Ti−O−Ti Complexes: Visible‐Light‐Activated, Homogeneous Alternative to TiO2 Photosensitisers
Source: Chemistry. 2020 Jul 9;26(43):9486–94. doi: 10.1002/chem.202001678 (PMC7496837; doi:10.1002/chem.202001678)
Supplement: Supplementary file 1 — Supplementary [file CHEM-26-9486-s001.pdf]

Chemistry–A European Journal

Supporting Information

Discrete Ti—O—Ti Complexes: Visible-Light-Activated, Homogeneous Alternative to TiO_2 Photosensitisers

Kira Behm, Eszter Fazekas, Martin J. Paterson, Filipe Vilela, and Ruaraidh D. McIntosh*[a]

Supporting Information

Table of Contents

1. NMR Data 2

1.1 ^{1}H NMR spectra of pro-ligands L1H_2 – L4H_2 2

1.2 ^{1}H and ^{13}C NMR spectra of complexes C1 – C5 4

2. ESI Mass Spectrometry Data 8

3. UV-vis Data of a Pro-ligand 9

4. $^1O_2$ Flow Experiments 10

5. Crystal Data 12

6. References 13

1. NMR Data

Pro-ligands **L1H$_2$**,[1] **L2H$_2$**,[2] **L3H$_2$**[3] and **L4H$_2$**[4] were published previously and the recorded spectra were in accordance with the reported data.

1.1 ^{1}H NMR spectra of pro-ligands L1H$_2$ – L4H$_2$

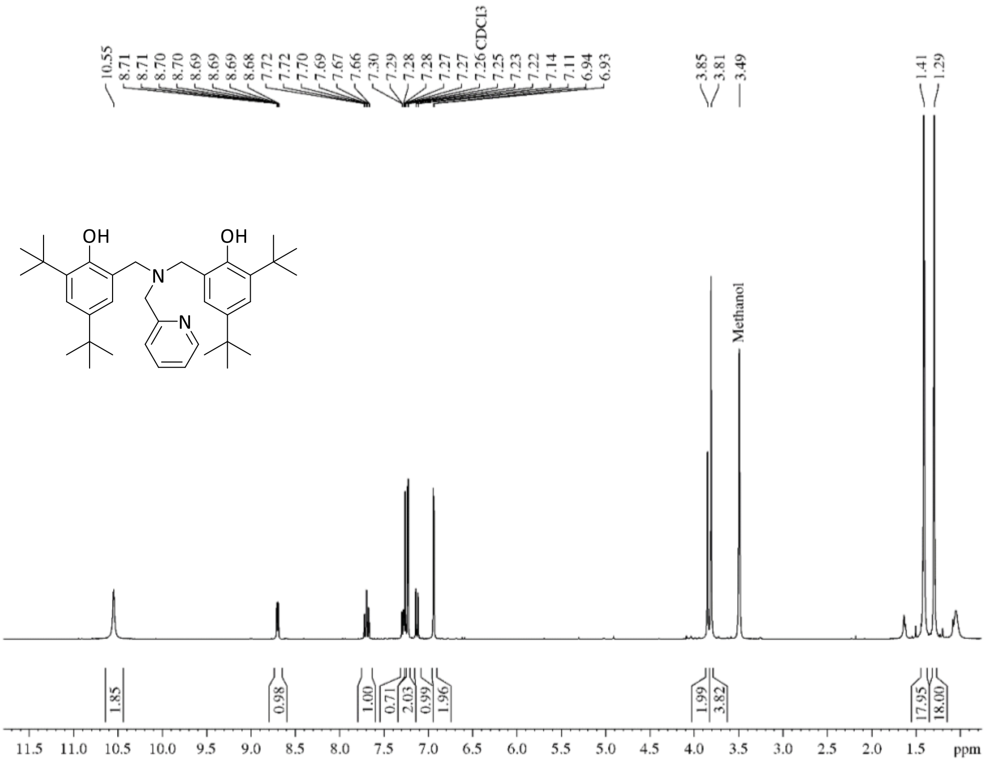

*Figure S1: ^{1}H NMR spectrum of pro-ligand **L1H$_2$** in $CDCl_3$ at 25°C.*

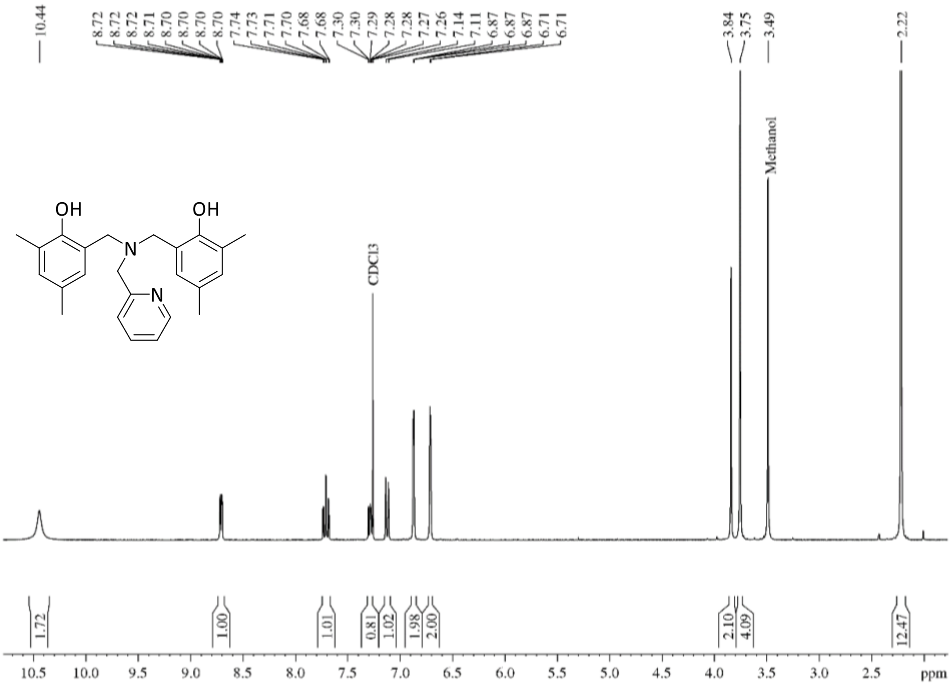

*Figure S2: ^{1}H NMR spectrum of pro-ligand **L2H$_2$** in $CDCl_3$ at 25°C.*

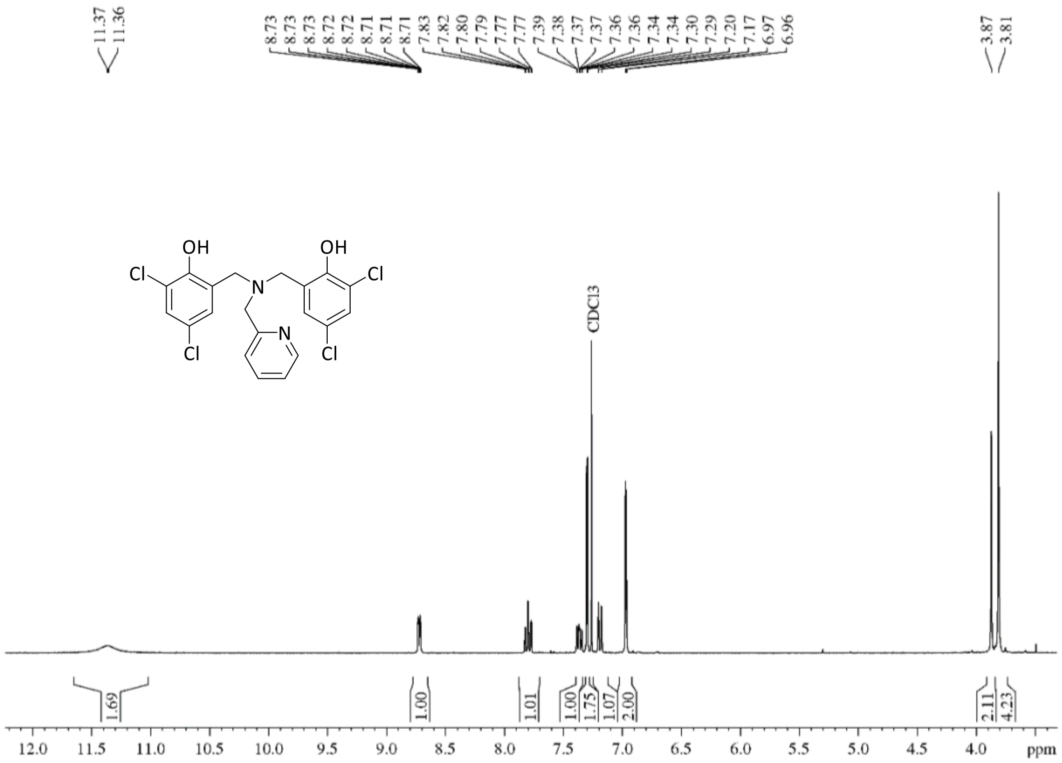

*Figure S3: ^{1}H NMR spectrum of pro-ligand **L3H$_2$** in CDCl$_3$ at 25°C.*

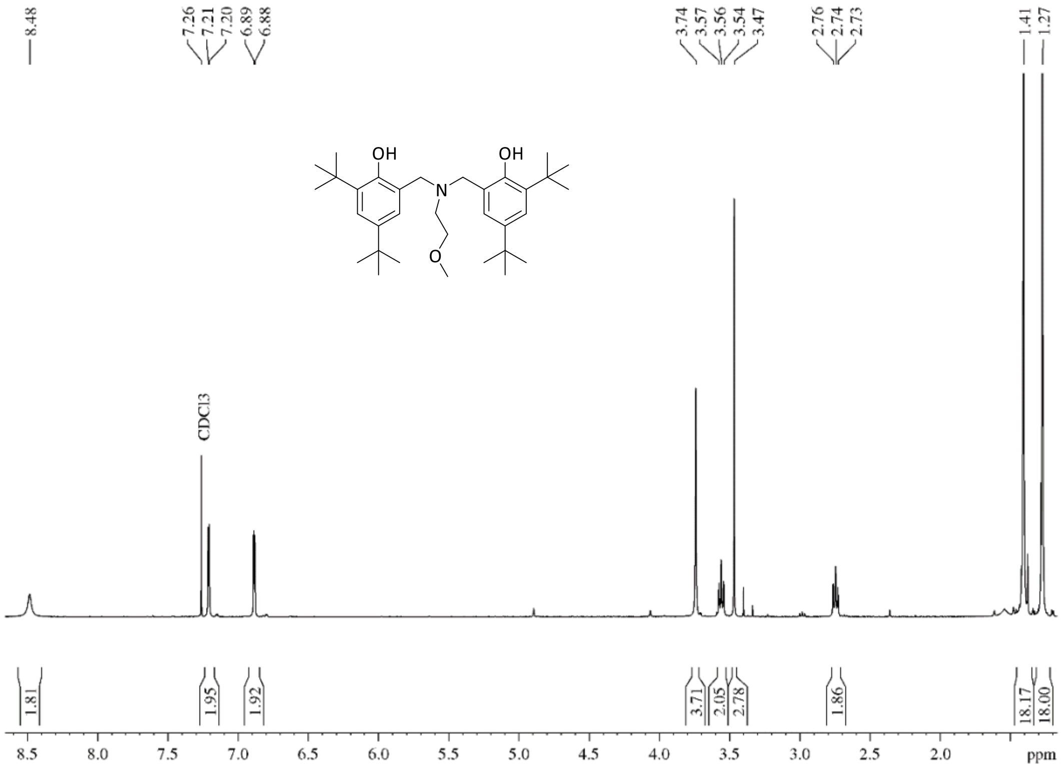

*Figure S4: ^{1}H NMR spectrum of pro-ligand **L4H$_2$** in CDCl$_3$ at 25°C.*

1.2 ^{1}H and ^{13}C NMR spectra of complexes **C1** – **C5**

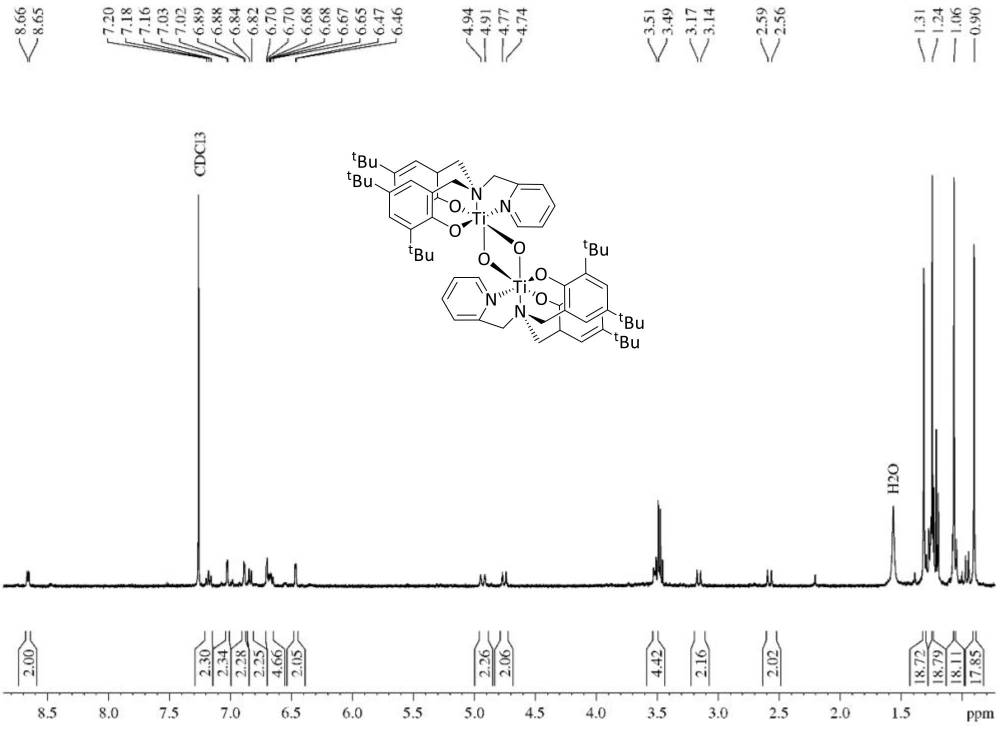

*Figure S5: ^{1}H NMR spectrum of complex **C1** in $CDCl_3$ at 25°C.*

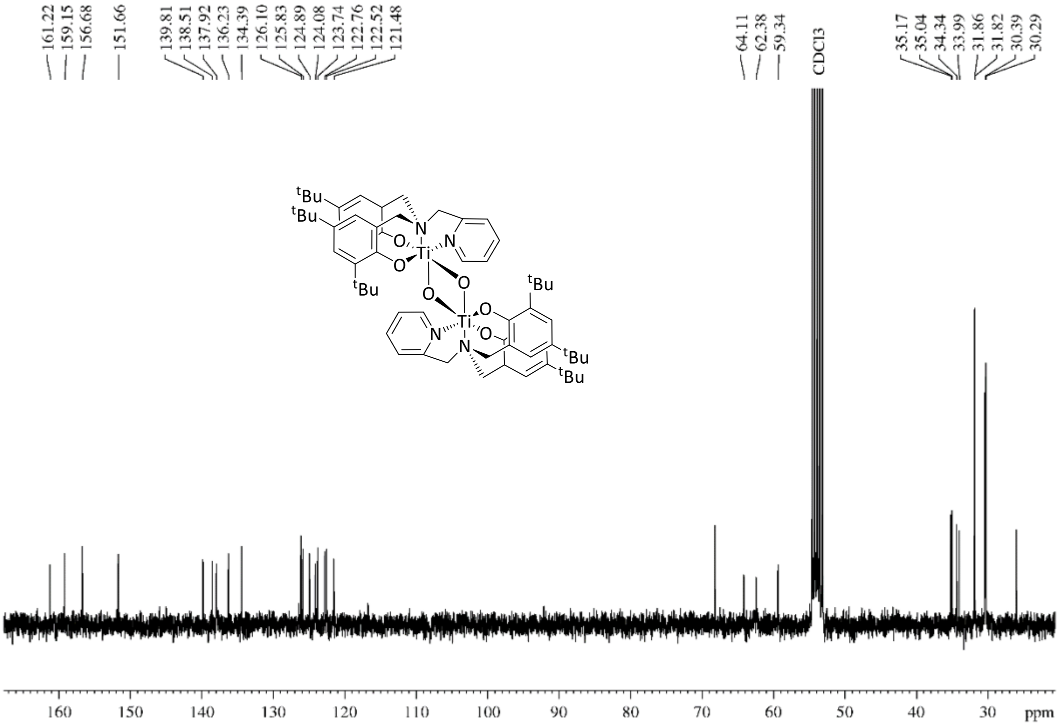

*Figure S6: ^{13}C NMR spectrum of complex **C1** in CD_2Cl_2 at 25°C.*

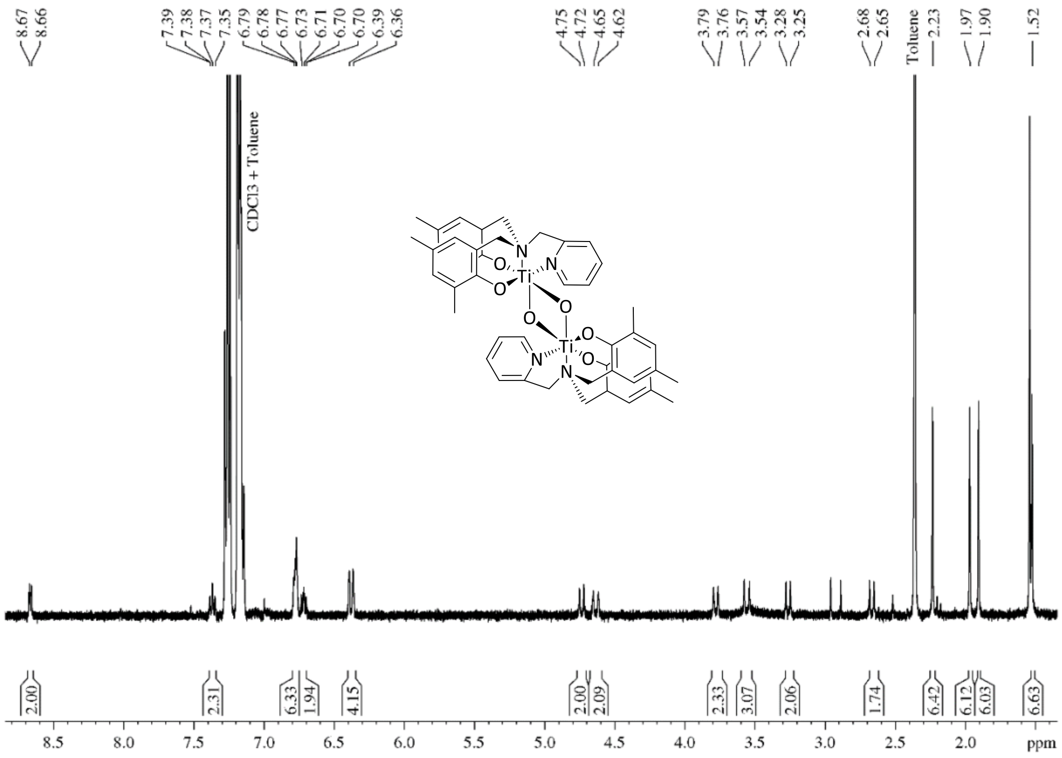

*Figure S7: ^{1}H NMR spectrum of complex **C2** in $CDCl_3$ at 25°C.*

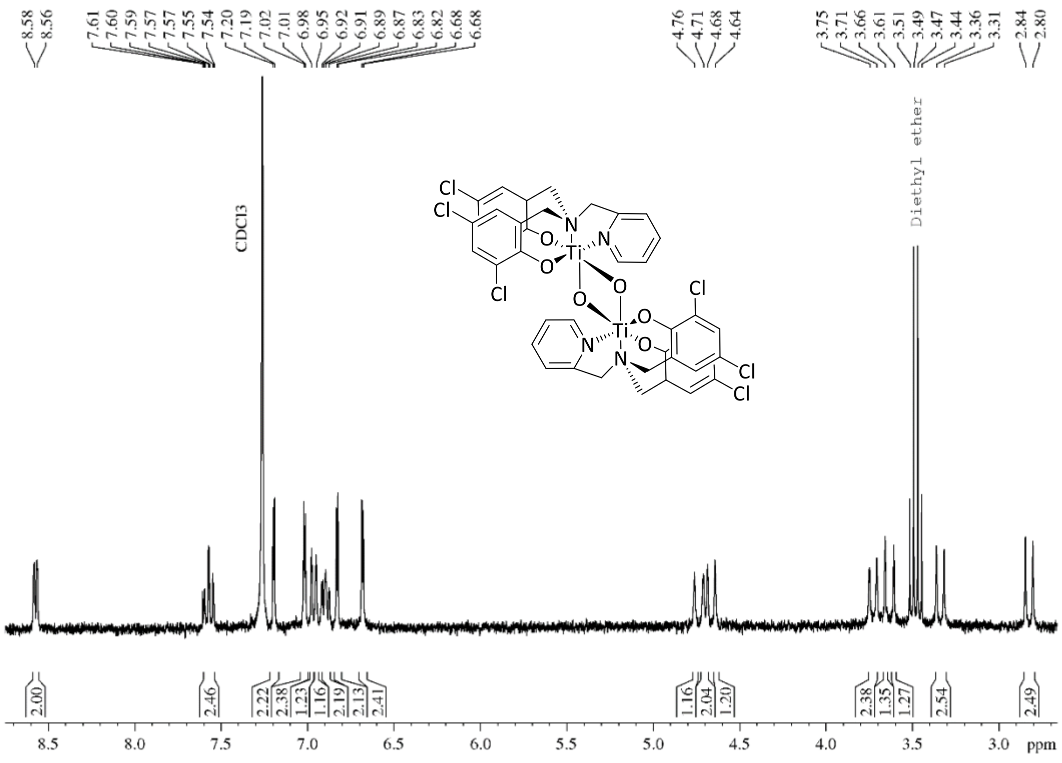

*Figure S8: ^{1}H NMR spectrum of complex **C3** in $CDCl_3$ at 25°C.*

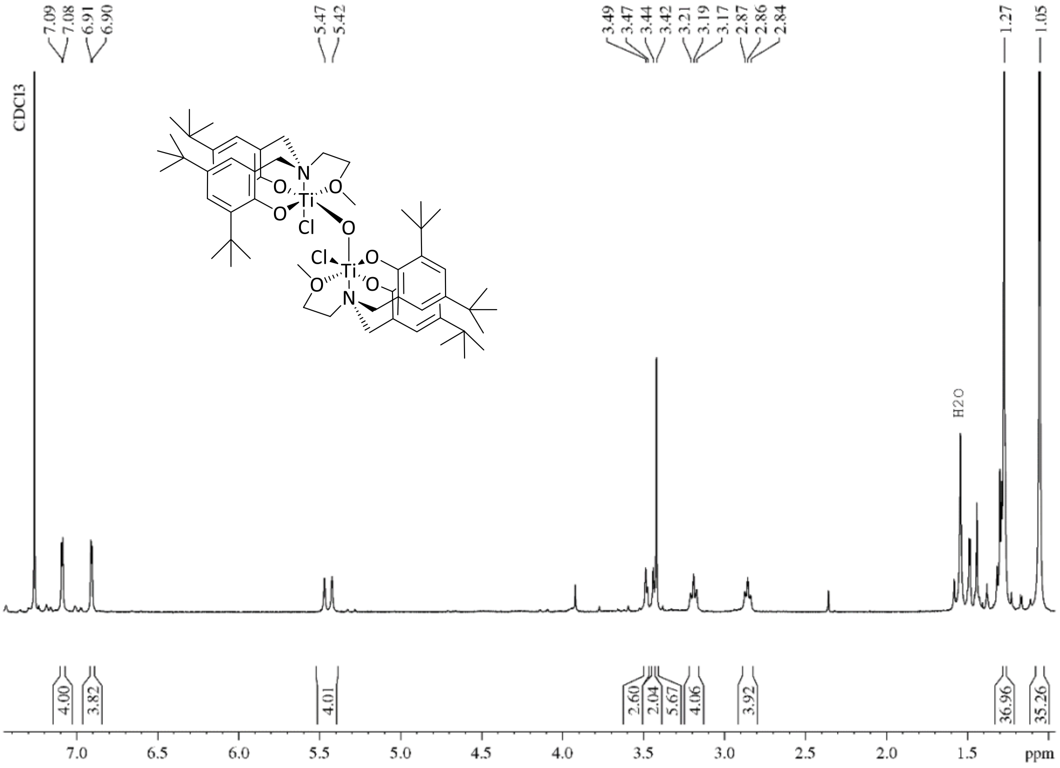

*Figure S9: ^{1}H NMR spectrum of complex **C4** in $CDCl_3$ at 25°C.*

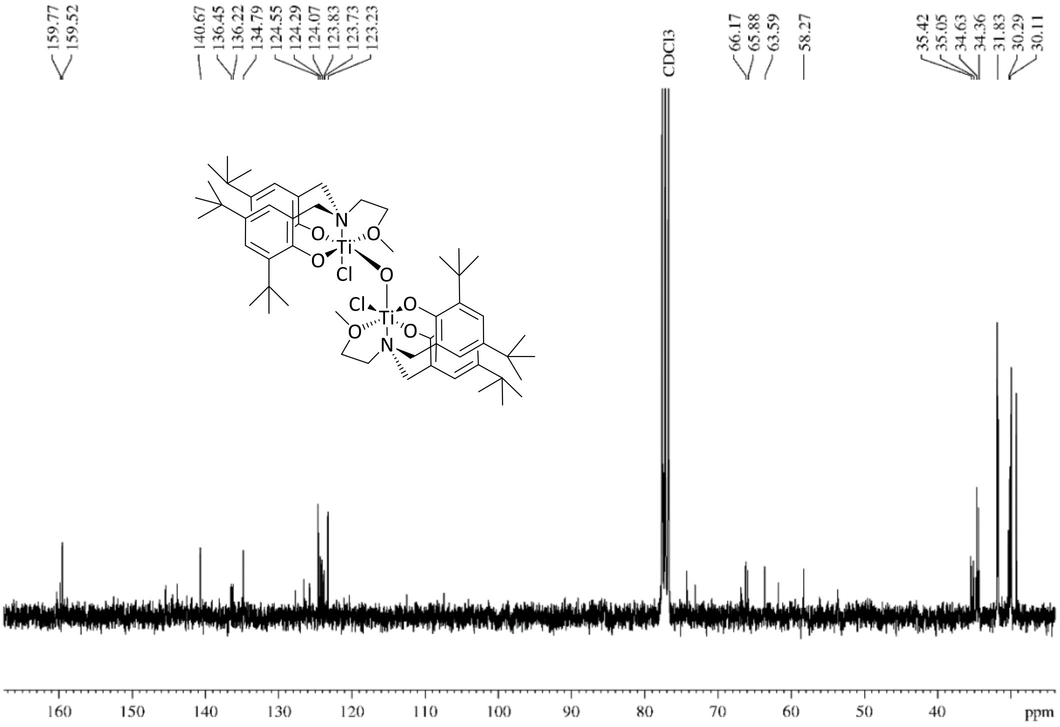

*Figure S10: ^{13}C NMR spectrum of complex **C4** in $CDCl_3$ at 25°C.*

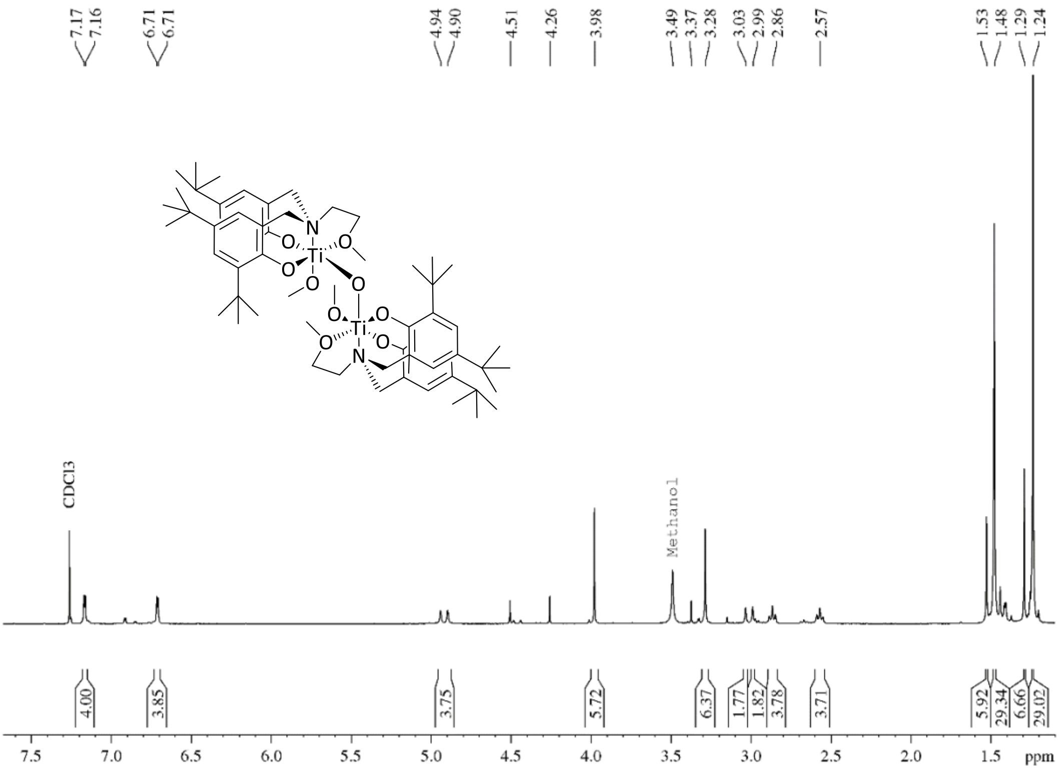

Figure S11: ^{1}H NMR spectrum of complex ***C5*** *in $CDCl_3$ at 25°C.*

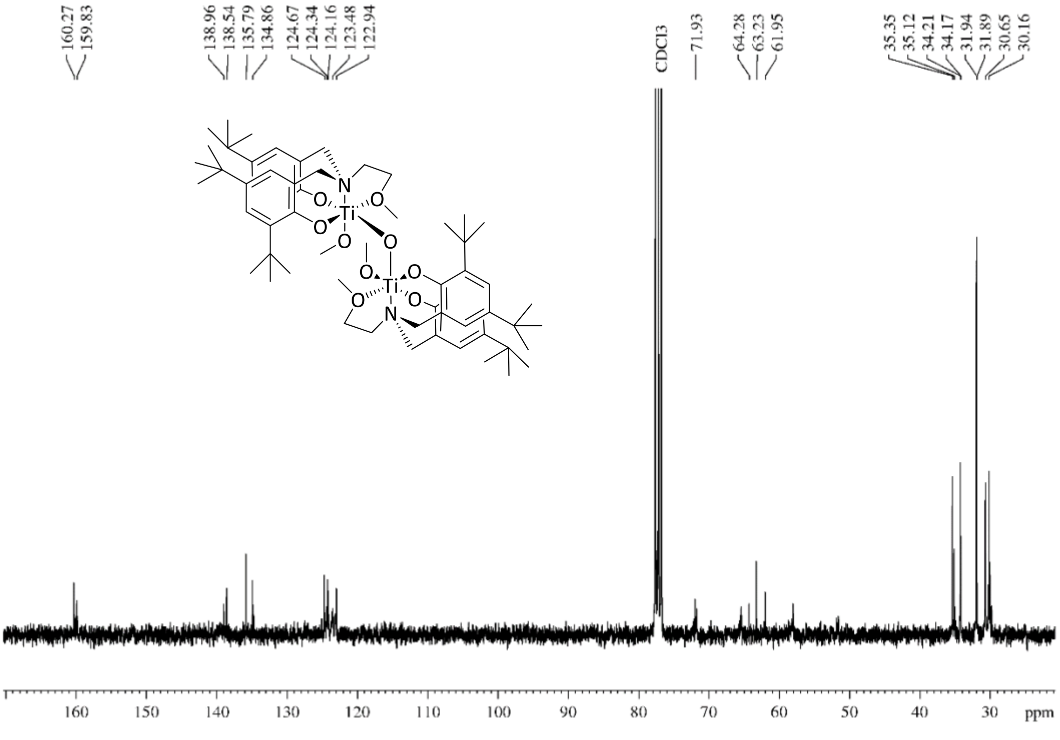

Figure S12: ^{13}C NMR spectrum of complex ***C5*** *in $CDCl_3$ at 25°C.*

2. ESI Mass Spectrometry Data

C1

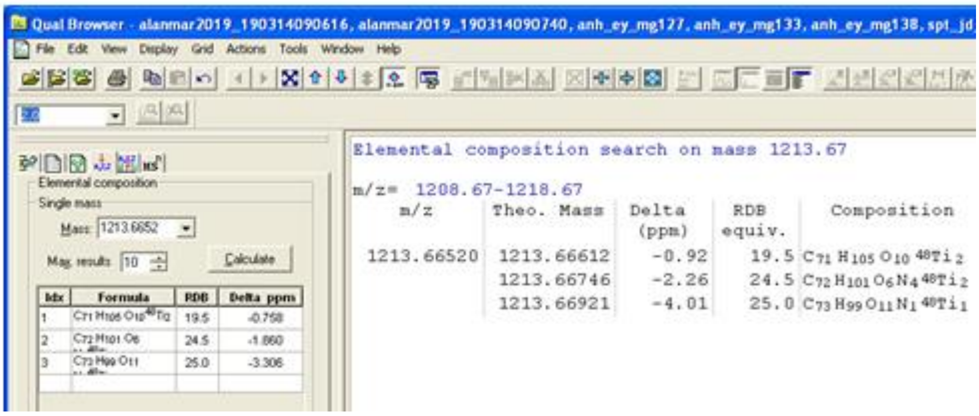

*Figure S13: Accurate mass analysis for **C1**.*

C2

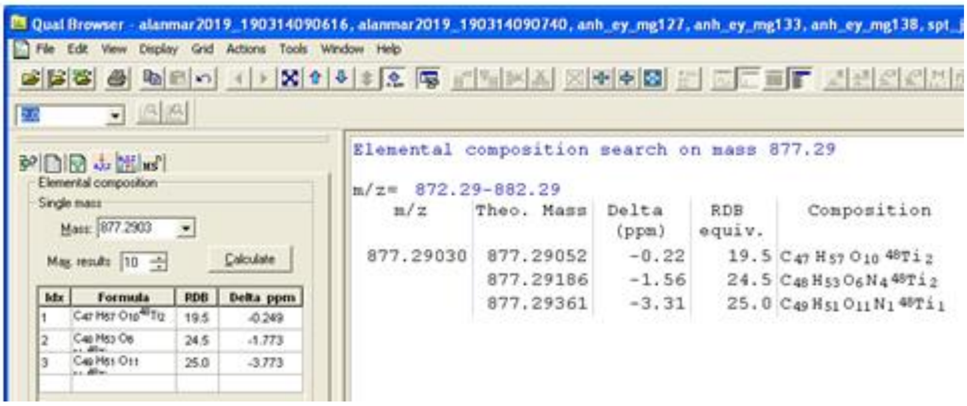

*Figure S14: Accurate mass analysis for **C2**.*

C3

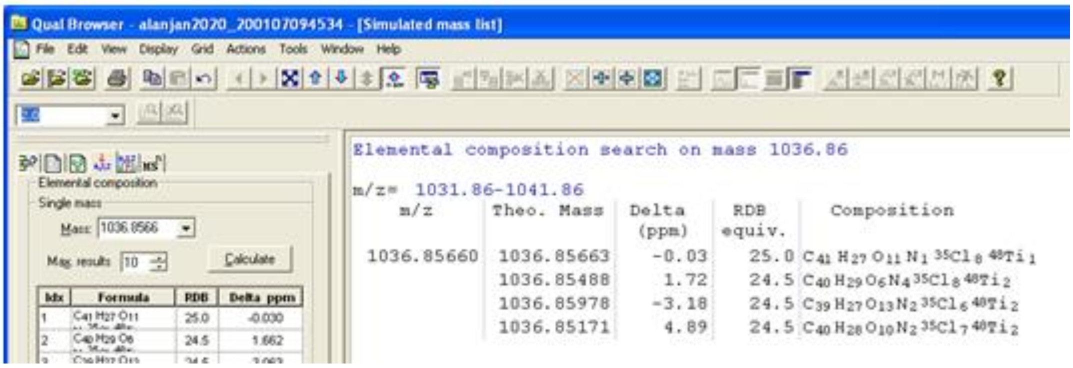

*Figure S15: Accurate mass analysis for **C3**.*

C4

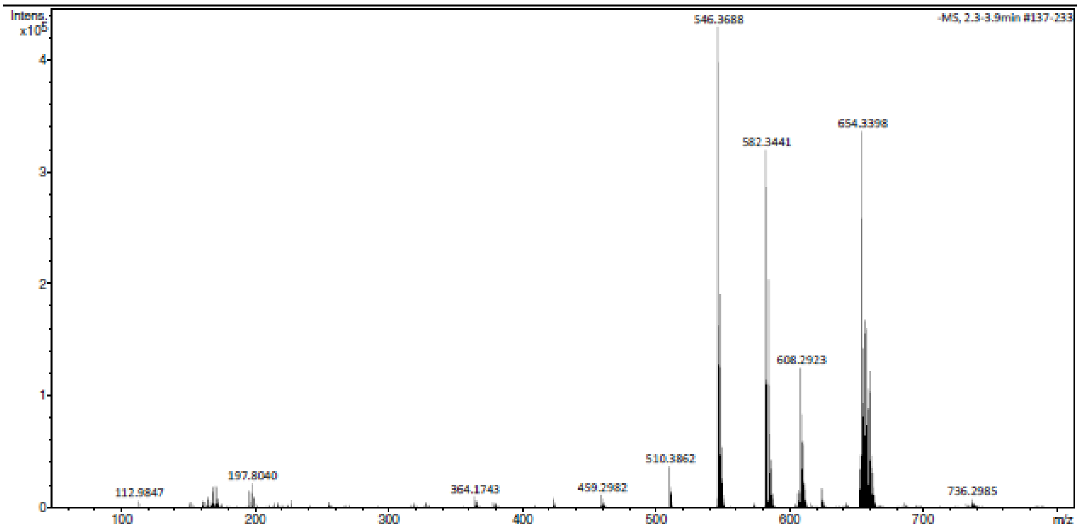

Figure S16: Mass spectrometry analysis for ***C4****.*

C5

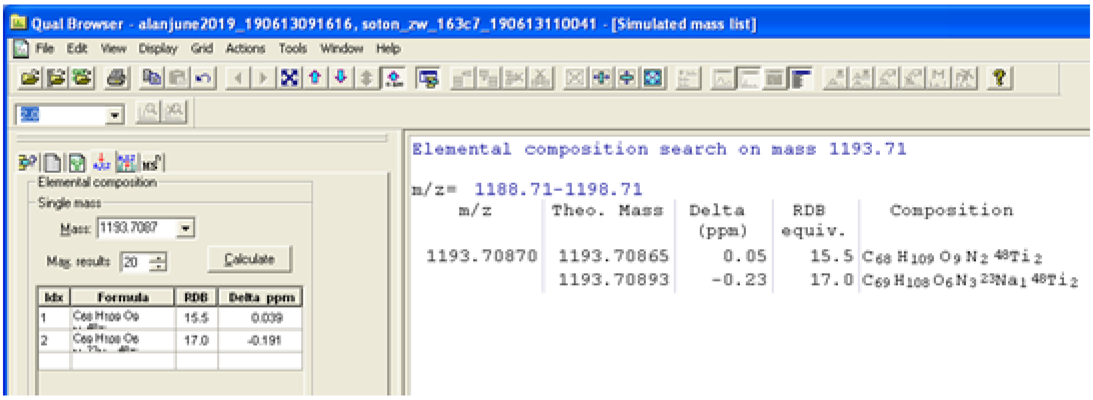

Figure S17: Accurate mass analysis for ***C5****.*

3. UV-vis Data of a Pro-ligand

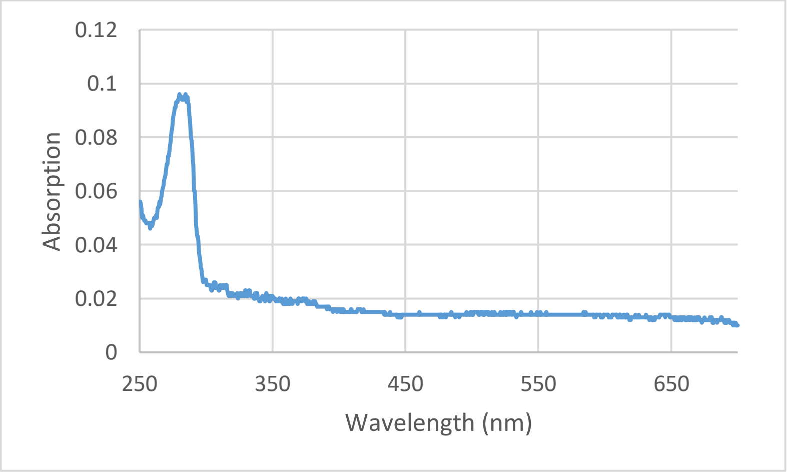

Figure S18: UV-vis spectrum of pro-ligand ***L4H₂****.*

4. $^1O_2$ Flow Experiments

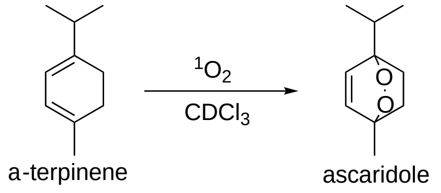

Scheme S1: Photooxygenation of α-terpinene to ascaridole.

Control experiments in flow were conducted with complex **C4** ($9.5*10^{-3}$ mmol) and α-terpinene (0.19 mmol). Without irradiation, no conversion was observed after 2.5 hours. Without mixing with O_2, but still allowing air contact, a conversion of 25 % was observed in three hours. The α-terpinene was found to slightly self-sensitise under irradiation at 420 nm and yielded 4 % conversion to ascaridole in three hours without the addition of a catalyst. Pro-ligand **$L4H_2$** on its own yielded 20 % conversion in six hours. Initial experiments were carried out both with and without using 1,3,5-methoxybenzene as an internal standard to ensure the α-terpinene did not evaporate over the course of the reaction. It was found that the loss of α-terpinene stayed below 10 % (i.e. below the standard error of NMR integration). Therefore, further experiments were conducted without the addition of internal standards.

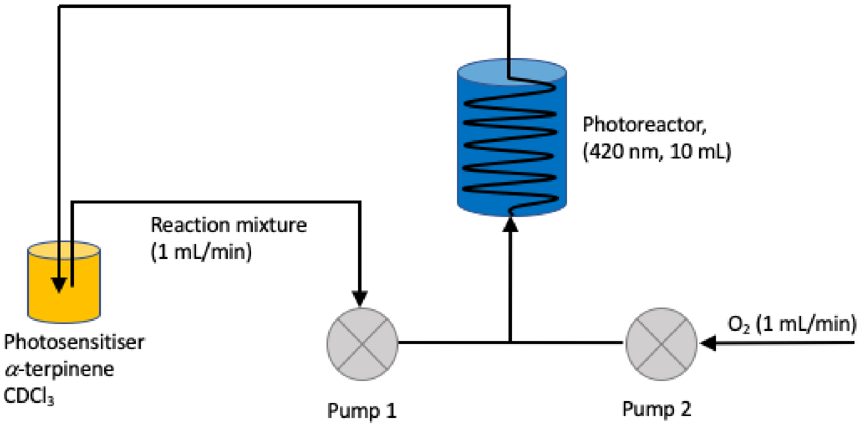

Figure S19: Schematic of experimental setup of the Vapourtec flow reactor.

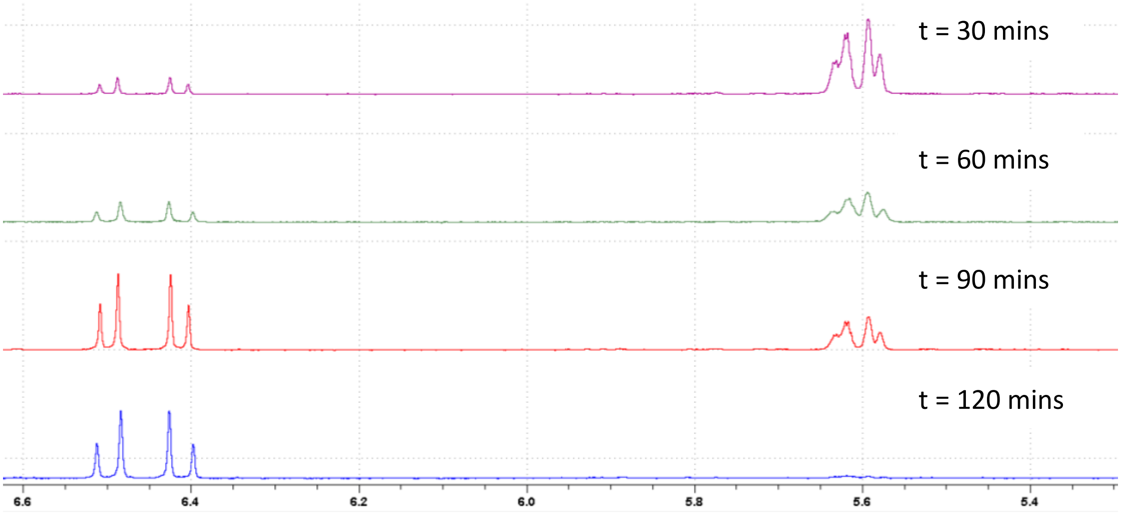

Figure S20: ^{1}H NMR trace of α-terpinene (right) to ascaridole (left) conversion.[5]

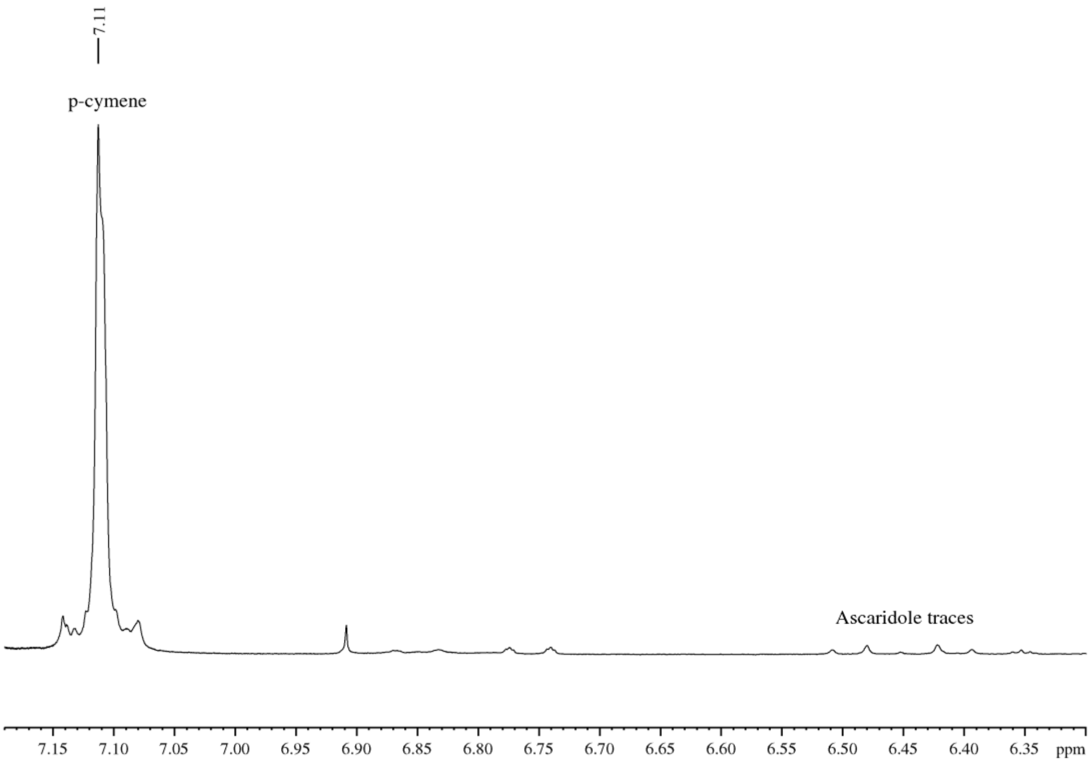

Figure S21: Appearance of p-cymene peak at 7.11 ppm in control experiment with TiO_2.[6-7]

5. Crystal Data

	C2	***C3***	***C4***	***C5***
CCDC Code	1992707	1992708	1992709	1992710
Empirical formula	$C_{24}H_{27}N_2O_3Ti$	$C_{20}H_{14}Cl_4N_2O_3Ti$	$C_{66}H_{102}Cl_2N_2O_7Ti_2$	$C_{68}H_{108}N_2O_9Ti_2$
Formula weight	439.37	520.03	1202.19	1193.35
Temperature/K	120.01(10)	120.01(10)	100.0	100.0
Crystal system	trigonal	triclinic	monoclinic	monoclinic
Space group	R-3	P-1	$P2_1/c$	$P2_1/n$
a/Å	32.5530(11)	7.8843(2)	16.1685(6)	23.3829(6)
b/Å	32.5530(11)	11.9265(4)	11.5807(4)	13.0475(3)
c/Å	12.9542(5)	13.0653(5)	17.4316(5)	24.3364(6)
α/°	90	79.281(3)	90	90
β/°	90	74.536(3)	90.6268(17)	112.893(2)
γ/°	120	85.952(3)	90	90
Volume/$Å^3$	11888.4(9)	1163.14(7)	3263.74(19)	6839.9(3)
Z	23	2	2	4
$\rho_{calc}g/cm^3$	1.412	1.485	1.223	1.376
μ/mm^{-1}	0.443	0.851	3.233	4.326
F(000)	5313.0	524.0	1292.0	2960.0
Crystal size/mm^3	0.365 × 0.247 × 0.212	0.282 × 0.216 × 0.104	0.18 × 0.16 × 0.06	0.20 × 0.15 × 0.04
Radiation	MoKα (λ = 0.71073)	MoKα (λ = 0.71073)	CuKα (λ = 1.54178)	CuKα (λ = 1.54178)
2Θ range for data collection/°	6.086 to 57.592	6.142 to 59.336	9.168 to 149.226	4.448 to 96.998
Index ranges	-36 ≤ h ≤ 43, -43 ≤ k ≤ 40, -16 ≤ l ≤ 15	-10 ≤ h ≤ 10, -16 ≤ k ≤ 15, -18 ≤ l ≤ 17	-20 ≤ h ≤ 20, -14 ≤ k ≤ 13, -21 ≤ l ≤ 21	-22 ≤ h ≤ 22, -12 ≤ k ≤ 12, -23 ≤ l ≤ 23
Reflections collected	42184	21157	64722	44476
Independent reflections	6451 [R_{int} = 0.0636, R_{sigma} = 0.0472]	5813 [R_{int} = 0.0367, R_{sigma} = 0.0404]	6623 [R_{int} = 0.1261, R_{sigma} = 0.0664]	6511 [R_{int} = 0.0748, R_{sigma} = 0.0510]
Data/restraints/parameters	6451/0/275	5813/0/271	6623/15/402	6511/87/807
Goodness-of-fit on F^2	1.037	1.020	1.053	1.048
Final R indexes [I>=2σ (I)]	R_1 = 0.0458, wR_2 = 0.1029	R_1 = 0.0364, wR_2 = 0.0795	R_1 = 0.0496, wR_2 = 0.1251	R_1 = 0.0643, wR_2 = 0.1294
Final R indexes [all data]	R_1 = 0.0598, wR_2 = 0.1081	R_1 = 0.0466, wR_2 = 0.0848	R_1 = 0.0749, wR_2 = 0.1349	R_1 = 0.0984, wR_2 = 0.1500
Largest diff. peak/hole / e $Å^{-3}$	0.31/-0.35	0.50/-0.49	0.64/-0.41	0.55/-0.43

6. References

[1] Y. Shimazaki, S. Huth, A. Odani, O. Yamauchi, *Angew. Chem. Int. Ed.* **2000**, *39*, 1666-1669.

[2] L.-Z. Fu, L.-L. Zhou, Q.-N. Liang, C. Fang, S.-Z. Zhan, *Polyhedron* **2016**, *107*, 83-88.

[3] A. M. Reckling, D. Martin, L. N. Dawe, A. Decken, C. M. Kozak, *J. Organomet. Chem.* **2011**, *696*, 787-794.

[4] E. Y. Tshuva, S. Groysman, I. Goldberg, M. Kol, *Organometallics* **2002**, *21*, 662-670.

[5] J. M. Tobin, T. J. D. McCabe, A. W. Prentice, S. Holzer, G. O. Lloyd, M. J. Paterson, V. Arrighi, P. A. G. Cormack, F. Vilela, *ACS Catal.* **2017**, *7*, 4602-4612.

[6] K. S. Elvira, R. C. R. Wootton, N. M. Reis, M. R. Mackley, A. J. Demello, *ACS Sus. Chem. Eng.* **2013**, *1*, 209-213.

[7] O. Shvydkiv, K. Jähnisch, N. Steinfeldt, A. Yavorskyy, M. Oelgemöller, *Catal. Today* **2018**, *308*, 102-118.
